# The Complete Genome Sequence of *Fibrobacter succinogenes* S85 Reveals a Cellulolytic and Metabolic Specialist

**DOI:** 10.1371/journal.pone.0018814

**Published:** 2011-04-19

**Authors:** Garret Suen, Paul J. Weimer, David M. Stevenson, Frank O. Aylward, Julie Boyum, Jan Deneke, Colleen Drinkwater, Natalia N. Ivanova, Natalia Mikhailova, Olga Chertkov, Lynne A. Goodwin, Cameron R. Currie, David Mead, Phillip J. Brumm

**Affiliations:** 1 DOE Great Lakes Bioenergy Research Center, University of Wisconsin-Madison, Madison, Wisconsin, United States of America; 2 Department of Bacteriology, University of Wisconsin-Madison, Madison, Wisconsin, United States of America; 3 US Dairy Forage Research Center, U.S. Department of Agriculture-Agricultural Research Services (USDA-ARS), Madison, Wisconsin, United States of America; 4 Lucigen Corporation, Middleton, Wisconsin, United States of America; 5 DOE Joint Genome Institute, Walnut Creek, California, United States of America; 6 Biosciences Division, Los Alamos National Laboratory, Los Alamos, New Mexico, United States of America; 7 C5–6 Technologies, Middleton, Wisconsin, United States of America; Duke University Medical Center, United States of America

## Abstract

*Fibrobacter succinogenes* is an important member of the rumen microbial community that converts plant biomass into nutrients usable by its host. This bacterium, which is also one of only two cultivated species in its phylum, is an efficient and prolific degrader of cellulose. Specifically, it has a particularly high activity against crystalline cellulose that requires close physical contact with this substrate. However, unlike other known cellulolytic microbes, it does not degrade cellulose using a cellulosome or by producing high extracellular titers of cellulase enzymes. To better understand the biology of *F. succinogenes*, we sequenced the genome of the type strain S85 to completion. A total of 3,085 open reading frames were predicted from its 3.84 Mbp genome. Analysis of sequences predicted to encode for carbohydrate-degrading enzymes revealed an unusually high number of genes that were classified into 49 different families of glycoside hydrolases, carbohydrate binding modules (CBMs), carbohydrate esterases, and polysaccharide lyases. Of the 31 identified cellulases, none contain CBMs in families 1, 2, and 3, typically associated with crystalline cellulose degradation. Polysaccharide hydrolysis and utilization assays showed that *F. succinogenes* was able to hydrolyze a number of polysaccharides, but could only utilize the hydrolytic products of cellulose. This suggests that *F. succinogenes* uses its array of hemicellulose-degrading enzymes to remove hemicelluloses to gain access to cellulose. This is reflected in its genome, as *F. succinogenes* lacks many of the genes necessary to transport and metabolize the hydrolytic products of non-cellulose polysaccharides. The *F. succinogenes* genome reveals a bacterium that specializes in cellulose as its sole energy source, and provides insight into a novel strategy for cellulose degradation.

## Introduction

Herbivorous mammals are essential components of terrestrial ecosystems and are major participants in the global carbon cycle, as well as the foundation of animal agriculture. Much of the plant biomass consumed by herbivores is degraded by symbiotic microorganisms in the host digestive tract. This symbiotic interaction between plant-degrading microbial communities and their herbivorous hosts is perhaps best exemplified by ruminants such as domestic cattle. Plant biomass digestion occurs in the rumen, a specialized pregastric fermentative organ that can comprise up to one-sixth of the weight of the host animal [1]. The ruminal fermentation is characterized by an incomplete anaerobic digestion in which plant material is converted to a mixture of C2 to C6 volatile fatty acids (VFAs), some of which are produced via intermediates such as succinic and lactic acids. These VFAs are used by the host as its primary energy source. The ruminal microflora are also responsible for producing other metabolic products, including methane, carbon dioxide and microbial cells, the last of which are digested postruminally to supply a major portion of the protein requirements of the host.

An analysis of bacterial diversity in the rumen reveals many microbes capable of degrading plant cell components (cellulose, hemicelluloses, and starch), including members of the genera *Ruminococcus*, *Prevotella*, and *Butyrivibrio* [2]. One of the most highly cellulolytic of the ruminal microbes is *Fibrobacter succinogenes*, a Gram-negative bacterium originally classified in the phylum Bacteroidetes [3], but later resolved to its own unique phylum, Fibrobacteres [4]. Several studies employing quantitative PCR have revealed that in dairy cattle, *F. succinogenes* comprises several percent of the total bacterial 16 S rRNA genes, a proxy for relative population size within the prokaryotic community [5]. The microbial species composition of the rumen depends strongly on the animal model used and feed composition, and in some cases *F. succinogenes* is the predominant cellulolytic organism (reviewed in [6]).

Pure culture studies have shown that *F. succinogenes* is a highly cellulolytic mesophilic bacterium capable of growth on crystalline cellulose with a maximum specific growth rate constant of ∼0.076 h^−1^ [7]. Moreover, this species is a very effective competitor for cellodextrin products of cellulose hydrolysis [8], [9], and its ability to efflux cellodextrins produced by intracellular cellodextrin phosphorylase may contribute to the cross-feeding of other ruminal bacteria, both cellulolytic and non-cellulolytic [10]. The substantial cellulolytic capabilities of *F. succinogenes* appear related to its unique mode of hydrolysis. Like most anaerobic cellulolytic bacteria, *F. succinogenes* does not excrete significant amounts of cellulases into its environment, and degradation requires attachment of cells to the cellulose surface. However, this species does not appear to contain surface-bound cellulosomes or their signature features such as scaffoldins or dockerin-binding domains [11] that comprise the degradation apparatus of the most commonly studied cellulolytic anaerobes such as *Clostridium thermocellum* or the ruminococci [12]. In *F. succinogenes*, degradation of crystalline cellulose may be facilitated by a highly unusual orientation of the cells along the crystallographic axis of the degrading cellulose fiber [13], [14].

To gain a better understanding of the biology of *F. succinogenes*, we sequenced the genome of the type strain *Fibrobacter succinogenes* subsp. *succinogenes* S85 ATCC 19169^T^ (Henceforth *Fibrobacter succinogenes*) to completion. A metabolic reconstruction analysis of this genome reveals how *F. succinogenes* is physiologically adapted to the rumen environment. An analysis of its plant polysaccharide degrading machinery coupled with growth assays reveals that, while it has the ability to hydrolyze a wide variety of polysaccharides, it can only utilize cellulose and its hydrolytic products for growth. These data confirm that *F. succinogenes* is a metabolic specialist that mediates its cellulolytic lifestyle by removing plant cell wall hemicelluloses to gain access to cellulose. Given the recent interest in optimizing carbohydrate degradation for the production of biofuels, the genome sequence of *F. succinogenes* not only provides insight into its unique lifestyle, but is also a valuable resource for understanding microbial models of plant cell wall deconstruction.

## Results

### General features of the *F. succinogenes* genome

The *F. succinogenes* genome consists of a single, circular chromosome of 3,842,635 base pairs with a GC content of 48%, confirming a previous report on a *F. succinogenes* genomic map [15]. Gene prediction revealed 3,085 putative coding sequences, covering 90.76% of the genome, with an average length of 1,130 bp. A total of three rRNA operons were identified including three 5 S rRNAs, three 16 S rRNAs, and three 23 S rRNAs; in addition, 59 tRNAs covering all 20 protein amino acids were also recovered (GenBank accession CP001792.1). Comparison of the predicted proteins in *F. succinogenes* against a database containing the proteins from over 1,100 other sequenced microbial genomes revealed that 1,787 proteins (58%) could be assigned a putative function, 510 proteins (16.5%) were similar to those encoding hypothetical proteins, and the remaining 788 proteins (25.5%) had no significant similarity to any protein in the database, indicating that these may be genus- or species-specific proteins.

### Phylogenetic placement of *F. succinogenes*

**Figure 1:**
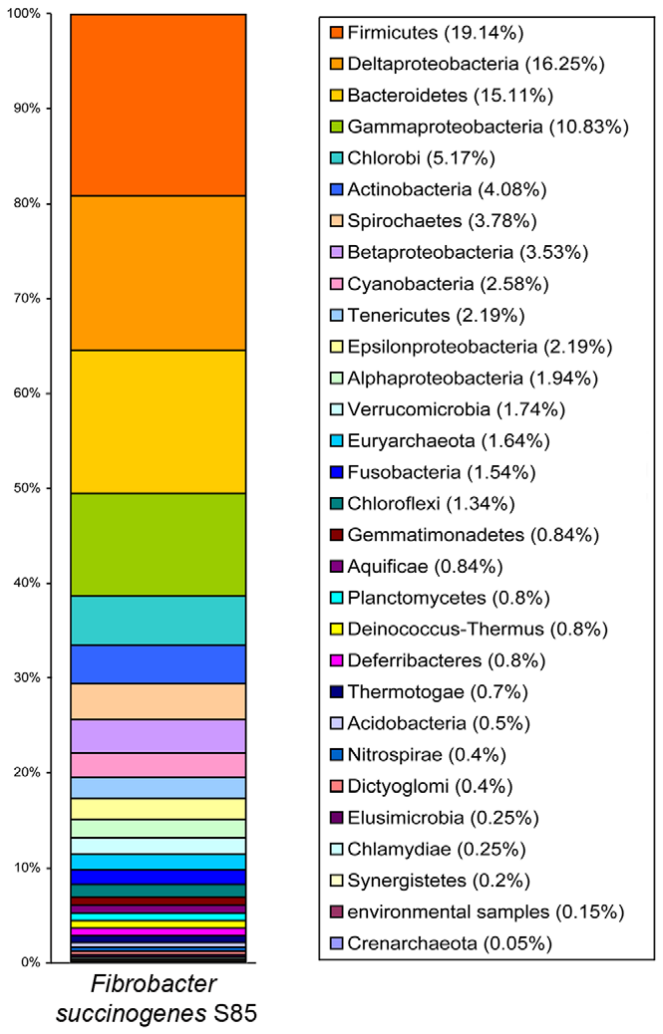
Taxonomic distribution analysis of the *F. succinogenes* proteome. The wide range of phyla/groups that *F. succinogenes* proteins are mapped to indicates it is not similar to other bacterial genomes, thereby confirming its placement into its own phylum.

*F. succinogenes* was originally placed within the phylum Bacteroidetes, and was included in the genus *Bacteroides* [3]. Subsequent 16 S rRNA sequencing revealed that *F. succinogenes* did not belong to the Bacteroidetes and was reclassified into the novel phylum Fibrobacteres [4]. Only the genus *Fibrobacter* has been described for this phylum, and this genus presently contains only one other formally described species, *F. intestinalis* [4]. Later work resolving the phylogenetic relationship of the Fibrobacteres relative to other phyla using housekeeping genes have shown that it is most closely related to the Bacteroidetes/Chlorobi [16], [17] and either the Actinobacteria [17] or the Chlamydiae [18]. To help further resolve this phylogenetic placement, we performed a taxonomic distribution analysis [19] by comparing each predicted protein in the *F. succinogenes* genome against a database containing all proteins from the complete microbial genome collection. For each *F. succinogenes* protein with a match, we determined the taxonomic identity of its top match and counted the total number of proteins in *F. succinogenes* that have their closest match to microbes belonging to a given phylum as shown in Figure 1. If *F. succinogenes* is closely related to bacteria in a different phylum, it is expected that a majority of proteins in *F. succinogenes* would have close matches to proteins in bacteria belonging to that phylum. We found that *F. succinogenes* contains proteins similar to a variety of bacteria in the current sequenced genome database collection. The majority of these proteins had multiple closest matches to bacteria belonging to Firmicutes (19%), Deltaproteobacteria (16%), Bacteroidetes (15%), Gammaproteobacteria (11%), and the Chlorobi (5%). These data confirm, on a whole-genome scale, that *F. succinogenes* belongs to its own phylum.

### COG analysis

To understand how *F. succinogenes* deploys genes in its genome, we performed a clusters of orthologous group (COGs) analysis [20]. This analysis places proteins into specific categories related to different aspects of cellular metabolism and physiology. A COG analysis of the *F. succinogenes* genome was able to classify 1,938 proteins into 17 different categories as shown in Table S1. From this analysis, over 30% of these proteins belong to four categories, including cell wall, membrane, and envelope biogenesis (category M, 11%); amino acid transport and metabolism (E, 9%); translation, ribosomal structure and biogenesis (J, 8%); and carbohydrate transport and metabolism (G, 8%). These results likely reflect the importance of these categories to the biology of *F. succinogenes*, as much of its lifestyle is dependent on the metabolism of carbohydrates for cellular processes. The high percentage of genes devoted to cell wall, membrane, and envelope biogenesis likely reflects *F. succinogenes* ability to adhere to plant biomass. In contrast, other categories have a paucity of proteins, including cell motility (N, 1%); secondary metabolites biosynthesis, transport and catabolism (Q, 2%); and defense mechanisms (V, 2%). The compositions of these categories are in accord with the lifestyle of *F. succinogenes*, as it is not actively motile and appears to have few defenses against antibiotics (see below). A key feature of the *F. succinogenes* COG analysis is the large number of proteins not identifiable: 1,147 out of 3,085 (37%) proteins could not be classified. Of the 1,938 proteins that could be classified, 357 (18%) were identified as either general function prediction only or function unknown. This indicates that over 50% of the *F. succinogenes* proteome could not be assigned to a known COG category. Although the COG database may not be extensive enough to capture all known functional categories, the large numbers of proteins with unassigned COGs, combined with our discovery that over 25% of the *F. succinogenes* proteome has no orthologs to microbes in the sequenced genome collection, may indicate numerous proteins unique to *F. succinogenes.*

### Polysaccharide degradation and utilization

A hallmark of *F. succinogenes* is its ability to efficiently hydrolyze the many plant polysaccharides it encounters in the rumen. We tested the ability of *F. succinogenes* to hydrolyze and/or utilize different polysaccharides by performing growth assays using a variety of substrates as shown in Table 1. Of the tested polysaccharides, which include cellulose, pectin, starch, glucomannan, arabinogalactan, and various forms of xylan, only cellulose was found to be both hydrolyzed and metabolized. A large number of other polysaccharides were found to be hydrolyzed without being metabolized, including all forms of xylan tested. The capacity to hydrolyze xylan but not utilize it for growth is reflected in *F. succinogenes*' genome, as it does not have the genes necessary to either transport or metabolize xylose or xylodextrins (see below). Therefore, the hydrolytic capability of this cellulose-dependent bacterium to degrade other plant polysaccharides suggests that *F. succinogenes* removes xylose-rich hemicelluloses to gain access to cellulose, and likely uses this exposed cellulose as its major energy source. We describe below two separate aspects of this strategy of plant cell wall deconstruction including the enzymatic hydrolysis of plant polysaccharides by each of the major classes of polysaccharide hydrolases and associated proteins, and the subsequent utilization of the products of cellulose hydrolysis. Other metabolic capabilities encoded by the genome are discussed in Supplemental Text including glycogen biosynthesis and utilization (Text S1), amino acid synthesis and nitrogen assimilation (Text S2), fatty acid synthesis and catabolism (Text S3), vitamin biosynthesis (Text S4), transporters (Text S5), antibiotic production and resistance (Text S6), DNA repair mechanisms (Text S7), and CRISPRs, insertion sequences, and genomic islands (Text S8).

**Table 1:** Polysaccharide degradation and utilization by *Fibrobacter succinogenes* S85.

Polysaccharide	Source	Linkage pattern^a^	mM succinate produced	mg reducing sugar accumulated /g added substrate
*Hydrolyzed and utilized:*				
Cellulose	Wood	β-1,4-Glc	20.6^b^	4.9^c^
*Hydrolyzed, but not utilized:*				
Homoxylan	Tobacco stalk	β-1,4-Xyl	0.1	381.8
Xylan	Larch	β-1,4-Xyl (with 4-*O*-MeGlcA substituents)	<0.1	328.6
Glucomannan	Salep	Mixed β-1,4-Glc and –Man	0.4	403.9
Xyloglucan	Tamarind	β-1,4-Glc (with β-1,6-Xyl substituents)	0	259.5
Pectin	Citrus	α-1,4-GalUA (with substantial methylation)	0.1	167.7
Inulin	Chicory	β-2,1-Fru	1.3	532
Phlein (Fructan)	Orchardgrass	β-2,6-Fru	0.3	13.8
*Not hydrolyzed or utilized*				
Arabinogalactan (type II)	(not specified)	β−1,3−Gal with some β−1,6−Gal sidechains capped with various monosaccharides	0.2	0
Curdlan	*Agrobacterium*	β−1,3−Glc	<0.1	0
Laminarin	Brown algae	β−1,3−Glc and β−1,6−Glc (mixed linkage, 3:1)	<0.1	0
Chitosan	Crab shells	β-1,4-GlcNH_2_ and *N*-AcGlcNH_2_	<0.1	0
Starch	Potato	α-1,4-Glc (with α-1,6 branches)	<0.1	0

a Monosaccharides and derivatives: Fru, fructose; Gal, galactose; GalUA, galacturonic acid, Glc, glucose; GlcNH2, glucosamine; Man, mannose; N-AcGlcNH2, *N*-acetylglucosamine; 4-*O*-MeGlcA, 4-*O*-methylglucuronic acid; Xyl, xylose.

b Mean of Avicel (20.6 mM), Amorphous cellulose (21.5 mM) and Cellulose II (19.9 mM).

c Mean of Avicel (6.2 mg/g), Amorphous cellulose (7.7 mg/g) and Cellulose II (0.8 mg/g).

### Enzymatic hydrolysis of polysaccharides

A carbohydrate-active enzyme (CAZy) [21] analysis revealed a total of 134 genes encoding enzymes that were classified into 49 different families of glycosyl hydrolases (GHs), carbohydrate binding modules (CBMs), carbohydrate esterases (CEs), and polysaccharide lyases (PLs) (Table S2). Because some of these enzymes have multiple CAZy family memberships (i.e. contain both a GH and a CBM), 196 total CAZymes were identified using this ontology. Only a small number of these CAZy family members have been experimentally characterized and these studies agree well with their bioinformatically predicted annotations, with some discrepancies [22]–[37] (Table S2). In addition, the majority of the *F. succinogenes* CAZymes are predicted to have signal peptides, indicating that these enzymes are targeted outside of the cytoplasm.

### Cellulases

*F. succinogenes* is predicted to possess 31 putative cellulase genes, including 10 members of GH5; 6 members of GH8; 9 members of GH9; 4 members of GH45; and 2 member of GH51 (Table 2). An analysis of the GHs predicted in the CAZy database for *C. thermocellum*, another prolific cellulose degrader, reveals a similar number of GH5 members, but only one member of GH8 and no members of GH45 or GH51. The GH45 members are especially significant, as these represent half of the predicted prokaryotic GH45 genes in the CAZy database. The most significant observation is that none of these 31 putative cellulase genes contain CBMs associated with binding to crystalline cellulose (CBM1, CBM2 or CBM3); in fact, the majority contains no recognizable CBM domains. A single xylanase-carboxymethyl cellulase has been reported to contain a GH5 and a CBM2 domain (AAC06197.1) [38], but BLAST analysis of this sequence reveals that it is not present in the *F. succinogenes* S85 genome. None of the cellulase genes contain domains that are homologous to known dockerin domains, and no genes with homology to scaffoldins were found in the genome, confirming the absence of cellulosomal structures in *F. succinogenes*. There was also no evidence of membrane anchoring or cell wall anchoring domains present in any of the cellulase genes. Further analysis of these cellulases using SignalP [39] reveals the presence of a signal peptide in many of these cellulases. Given the importance of adherence as an absolute requirement for cellulose degradation in this bacteria and the reported inability of the extracellular proteins produced by *F. succinogenes* to show cellulolytic activity [40], these cellulases may be targeted to one or more extra cytoplasmic environments where they could potentially interact with cellulose fibers.

**Table 2:** Known cellulases predicted from the *Fibrobacter succinogenes* S85 genome.

CAZy Family	Fisuc Locus	FSU Locus^a^	CBM Family	Signal Peptide	In Silico Prediction	Characterized Activity	Reference
GH5	Fisuc_0786	FSU_1228	–	Yes	cellulase	cellulase	This work
GH5	Fisuc_0897	FSU_1346	–	Yes	cellulase	cellulase	[24]
GH5	Fisuc_1224	FSU_1685	–	Yes	cellulase	cellulase	This work
GH5	Fisuc_1523	FSU_2005	–	Yes	cellulase	cellulase	This work
GH5	Fisuc_1584	FSU_2070	–	No	cellulase	–	[23]
GH5	Fisuc_1661	FSU_2150	–	Yes	endoglucanase	–	–
GH5	Fisuc_2011	FSU_2534	–	Yes	cellulase	–	–
GH5	Fisuc_2230	FSU_2772	–	Yes	endoglucanase	cellulase	[26]
GH5	Fisuc_2364	FSU_2914	–	Yes	cellulase	cellulase	[27]
GH5	Fisuc_3081	FSU_0347	–	Yes	cellulase	cellulase	This work
GH8	Fisuc_0207	FSU_0613	–	No	endoglucanase	xylanase	[22]
GH8	Fisuc_0241	FSU_0651	–	No	endoglucanase	cellulase	This work
GH8	Fisuc_0471	FSU_0889	–	Yes	endoglucanase	xylanase	[22]
GH8	Fisuc_1219	FSU_1680	–	Yes	endoglucanase	–	–
GH8	Fisuc_1802	FSU_2303	–	No	endoglucanase	cellulase	[27]
GH8	Fisuc_2579	FSU_3149	–	Yes	β-glucanase	cellulase	[22]
GH9	Fisuc_0057	FSU_0451	–	No	β-glucanase	cellulase	[25]
GH9	Fisuc_0393	FSU_0809	–	No	cellulase	–	–
GH9	Fisuc_0394	FSU_0810	–	No	cellulase	–	–
GH9	Fisuc_1531	FSU_2013	–	Yes	cellulase	–	–
GH9	Fisuc_1859	FSU_2361	–	Yes	β-glucanase	cellulase	[28]
GH9	Fisuc_1860	FSU_2362	–	Yes	cellulase	–	[28]
GH9	Fisuc_2033	FSU_2558	–	Yes	endoglucanase	cellulase	[29]
GH9	Fisuc_2362	FSU_2912	–	Yes	cellulase	cellulase	[30]
GH9	Fisuc_2876	FSU_0134	–	No	cellulase	–	–
GH45	Fisuc_1425	FSU_1893	–	No	cellulase	cellulase	[22]
GH45	Fisuc_1426	FSU_1894	–	No	cellulase	xylanase	[22]
GH45	Fisuc_1473	FSU_1947	–	Yes	cellulase	–	–
GH45	Fisuc_1933	FSU_2443	–	Yes	cellulase	–	–
GH51	Fisuc_2081	FSU_2610	–	Yes	endoglucanase	cellulase	[22]
GH51	Fisuc_3111	FSU_0382	CBM11, CBM30	Yes	β-glucanase	cellulase	[31]

a FSU locus tags refer to the equivalent ORF call in the *F. succinogenes* genome sequence project described by GenBank accession: CP002158.

Some of these predicted cellulases have been previously verified experimentally [22], [26]–[30] and we contributed to this growing list by performing cloning, expression, and carbohydrate hydrolysis assays on other putative glycoside hydrolases as shown in Table 2 and S2. We experimentally characterized a total of six cellulases including four GH5s, one GH8, and Fisuc_2005, which is predicted to belong to family PL10 but had cellulolytic activity in our assays (Table S2). The discovery of a PL10 with cellulolytic activity is perhaps surprising, but is commensurate with the reports of other GHs in *F. succinogenes* that have been described as having 'atypical' activities, including GH9 [29] and GH43 [41] enzymes.

An unanswered question regarding cellulose degradation by *F. succinogenes* is how these cellulase genes are regulated at the transcriptional level. In many polysaccharide-degrading bacteria, clusters of cellulases are often accompanied by transcriptional regulators that modulate their gene expression [42]–[45]. We analyzed clusters of genes surrounding cellulase-encoding genes in *F. succinogenes* and found only a handful had recognizable transcriptional regulators. These include an ArsR-type one-component regulator (Fisuc_0782) found upstream of a GH5 cellulase Fisuc_0786, a BadM-type transcriptional regulator (Fisuc_0900) found downstream of the GH5 cellulase Fisuc 0857, a sigma-24 extracellular cytoplasmic factor (ECF) transcriptional regulator (Fisuc_1517) found upstream of the GH5 cellulase Fisuc_1523, and a two-component sigma54-like response regulator (Fisuc_0397) found downstream of the GH9 cellulase Fisuc_0393. Any or all of these response regulators could potentially play a role in cellulase gene expression in *F. succinogenes*. Finally, a large number of cellulase (and hemicellulase) genes are found clustered between Fisuc_1762 and Fisuc 1804, which includes two metal-dependent response regulators and a sugar transporter. This cluster, which has been previously characterized [46], is likely controlled by these two response regulators, modulating the expression of both the GHs and the sugar transporter. Given the paucity of transcriptional regulators near known cellulase genes in *F. succinogenes*, it is likely that transcriptional regulators located in other portions of the genome contribute to the modulation of genes encoding for cellulases.

### *endo*-Hemicellulases

*F. succinogenes* possesses genes with a wide range of annotated hemicellulolytic activites including a large number of xylanases, arabinoxylanases, mannanases, curdlanases (β-1,3 glucanases), licheninases (β -1,4 glucanases), and xyloglucanases; these enzymes come from a range of GH families including GH10, GH11, GH18, and GH26 (Table 3). The annotated functions of these genes may not represent their true in vivo catalytic activities because of their low homology to characterized enzymes from other species. For example, when we cloned and expressed an annotated pectinase gene, Fisuc_0678, we found that it possessed cellulase and glucanase activity, but no pectinase activity (Table S2). To confirm the activity of other hemicellulases, we also characterized two GH26 β-mannanases (Fisuc_0727 and Fisuc_0729) as shown in Table 3.

**Table 3:** Known *endo*- and *exo*-hemicellulases predicted from the *Fibrobacter succinogenes* S85 genome.

CAZy Family	Fisuc Locus	FSU Locus^a^	CBM Family	Basic Terminal domain^b^	Signal Peptide	In Silico Prediction	Characterized Activity	Reference
GH2	Fisuc_1788	FSU_2288	–	BTD	Yes	β-galactosidase	–	–
GH2	Fisuc_3049	FSU_0315	–	–	No	β-galactosidase	β-galactosidase	This work
GH3	Fisuc_1751	FSU_2249	–	–	Yes	β-xylosidase	–	–
GH3	Fisuc_1985	FSU_2508	–	–	Yes	β-xylosidase	–	–
GH3	Fisuc_2065	FSU_2592	–	–	Yes	β-xylosidase	cellobiosidase	This work
GH10	Fisuc_0754	FSU_1192	–	–	Yes	xylanase	–	–
GH10	Fisuc_0757	FSU_1195	–	–	Yes	xylanase	–	–
GH10	Fisuc_1791	FSU_2292	CBM6	BTD	Yes	xylanase	–	[32]
GH10	Fisuc_1793	FSU_2293	CBM6	BTD	yes	xylanase	xylanase	[32]
GH10	Fisuc_1794	FSU_2294	CBM6	BTD	Yes	xylanase	xylanase	[32]
GH10	Fisuc_2303	FSU_2851	–	BTD	Yes	endoglucanase	–	–
GH10	Fisuc_2992	FSU_0257	–	–	Yes	cellulase	–	[33]
GH11	Fisuc_0362	FSU_0777	–	BTD	Yes	xylanase	xylanase	[34]
GH11	Fisuc_2201	FSU_2741	–	BTD	Yes	xylanase	xylanase	[22]
GH11	Fisuc_2442	FSU_3006	–	–	Yes	xylanase	xylanase	[22]
GH18	Fisuc_1530	FSU_2012	–	–	Yes	chitinase	cellulose binding	[35]
GH18	Fisuc_2465	FSU_3030	–	–	Yes	chitinase	–	–
GH26	Fisuc_0727	FSU_1165	CBM35	–	Yes	mannanase	β-mannanase	This work
GH26	Fisuc_0729	FSU_1167	CBM35	–	Yes	mannanase	β-mannanase	This work
GH26	Fisuc_0730	FSU_1168	–	–	Yes	mannanase	β-mannanase	[22]
GH26	Fisuc_1266	FSU_1729	–	–	Yes	mannanase	–	–
GH26	Fisuc_1688	FSU_2181	CBM35	–	Yes	mannanase	–	
GH43	Fisuc_1762	FSU_2262	CBM6	BTD	No	β-xylosidase	–	–
GH43	Fisuc_1763	FSU_2263	CBM6	BTD	Yes	β-xylosidase	–	–
GH43	Fisuc_1764	FSU_2264	CBM6	BTD	Yes	β-xylosidase	–	[22]
GH43	Fisuc_1769	FSU_2269	CBM6	BTD	No	β-xylosidase	arabinoxylanase	This work
GH43	Fisuc_1775	FSU_2274	CBM6	BTD	Yes	β-xylosidase	–	–
GH43	Fisuc_1994	FSU_2517	CBM35, CBM61	–	Yes	β-xylosidase	arabinase	This work
GH43	Fisuc_1997	FSU_2520	–	–	No	xylanase	–	–
GH43	Fisuc_1998	FSU_2521	–	–	Yes	β-xylosidase	–	–
GH43	Fisuc_1999	FSU_2522	CBM35	–	No	β-xylosidase	–	–
GH43	Fisuc_2621	FSU_3190	–	–	Yes	β-xylosidase	–	–
GH43	Fisuc_2622	FSU_3191	CBM35	–	Yes	β-xylosidase	–	–
GH43	Fisuc_2623	FSU_3192	CBM35	–	Yes	β-xylosidase	–	–
GH43	Fisuc_2886	FSU_0145	–	–	Yes	β-xylosidase	–	–
GH43	Fisuc_2929	FSU_0192	CBM6	BTD	No	xylanase	–	–

a FSU locus tags refer to the equivalent ORF call in the *F. succinogenes* genome sequence project described by GenBank accession: CP002158.

b Basic Terminal Domain (BTD) is also known as *F. succinogenes*-specific paralogous module 1 (FPm-1) [46].

Our hydrolysis and utilization assays reveal *F. succinogenes* cannot metabolize any products produced by these *endo*-hemicellulases (Table 1), and this is further underscored by the lack of genes necessary to both transport and metabolize these carbohydrates. For example, *F. succinogenes* is missing the genes necessary for xylose and xylodextrin transport (i.e. a xylose permease) into the cell, and further lacks a xylose isomerase (EC 5.3.1.5) to convert xylose into xylulose. As a result, we surmise that the function of these *endo*-hemicellulases is to serve as a form of biomass pretreatment by removing hemicelluloses and making cellulose microfibrils accessible to attack by the organism. Unlike the cellulases, many of the hemicellulases (GH10 and GH26) contain CBM6 or CBM35 domains that are known to bind to single chain substrates. These may increase the activity of the enzymes on the insoluble hemicellulose substrates present in the cell walls of biomass. Recently a novel domain, designated *F. succinogenes*-specific paralogous module 1 (FPm-1) was identified in 24 genes of *F. succinogenes* [46]. The function of this positively charged, basic domain located at the C-terminus of the proteins is unclear, but may be related to transport and localization of these proteins to the outer surface of the cell, or be involved in binding of the proteins to the outer membrane.

In general, the *endo*-hemicellulases, like the cellulases, contain a majority of enzymes with signal peptides. This fits with a general model of *F. succinogenes* hemicellulases localized to the outer membrane, which would facilitate the removal of these carbohydrates to gain access to cellulose.

### *exo*-Hemicellulases

One would expect that an organism that is unable to metabolize the monosaccharides released by *exo*-hemicellulases would possess few of these genes. Yet *F. succinogenes* possesses an unexpectedly large number of *exo*-acting hemicellulolytic enzymes: 2 members of GH2; 3 members of GH3; and 11 members of GH43 (Table 3). Like the *endo*-hemicellulases, many of the hemicellulases (GH43) contain CBM6 domains, known to bind to single chain substrates. These GH43 family members contain β-xylosidases and α-arabinofuranosidases; the CBM6 regions may improve synergistic interactions of the GH43 enzymes with the *endo*-hemicellulases for degradation of insoluble substrates. The function of these enzymes may be to provide growth substrates for the microbial consortia present in the rumen that supplies the essential branched and odd-chain length volatile fatty acids needed by *F. succinogenes.* Surprisingly, *F. succinogenes* appears not to contain any putative *alpha*-glucuronidase genes. This suggests that the specificities of *F. succinogenes endo*-hemicellulases and *exo-*hemicellulases may be such that they are able to bypass *alpha*-glucuronic acid residues in substituted xylans. Alternately, the organism may utilize an unrecognized gene product with *alpha*-glucuronidase to perform the hydrolysis. In addition to cataloguing these *exo*-hemicellulases, we characterized and verified the activity of two GH43 enzymes, including Fisuc_1769 as an arabinoxylanase and Fisuc_1994 as an arabinase (Table 3).

Like the cellulases and *endo*-hemicellulases, almost all of the *exo-*hemicellulases contain signal peptides and thus are also secreted from the cytoplasm. These data, along with our carbohydrate utilization assays (Table 1) support the model that *F. succinogenes* is a cellulose-degrading specialist. The localization and interactions between *exo*- and *endo*-hemicellulases at the cell membrane would facilitate the removal of hemicellulose and provide *F. succinogenes* with direct access to cellulose, which it actively degrades and metabolizes.

### Carbohydrate esterases

In addition to the large number of *endo*- and *exo-*hemicellulases present in the *F. succinogenes* genome, the genome sequence also predicts genes for 17 CEs from families CE1, CE2, CE6, CE8, CE12, and CE15 (Table 4). All but 3 of the 17 CEs are predicted to have signal peptides, indicating their secretion outside the cytoplasm. Like the *exo-*hemicellulases, many of these genes (8 of 17) contain CBM modules that may assist in binding to and degrading of insoluble hemicelluloses and pectins. The *in vivo* substrates of these individual CE enzymes are unclear, because many of these genes show low homology to known and characterized esterases. The presence of CBM modules and signal peptides suggest these proteins may act synergistically with *endo*-hemicellulases and *exo-*hemicellulases in the degradation of xylan and pectin by cleaving acetic and ferulic acid esters linkages. A number of these enzymes have been characterized [47]–[49], and *in vitro* activity has been demonstrated on synthetic substrates such as p-nitrophenyl acetate, xylose tetraacetate, or glucose pentacetate. In addition to cataloguing these CEs, we characterized and verified the activity of three putative esterases including a CE1 (Fisuc_1771), a CE2 (Fisuc_1641), and a CE6 (Fisuc_2534). All were found to possess acetyl esterase activity on p-nitrophenyl acetate (Table 4).

**Table 4:** Known carbohydrate esterases predicted from the *Fibrobacter succinogenes* S85 genome.

CAZy Family	Fisuc Locus	FSU Locus^a^	CBM Family	Basic Terminal domain^b^	Signal Peptide	In Silico Prediction	Characterized Activity	Reference
CE1	Fisuc_1771	FSU_2270	CBM4	BTD	Yes	esterase	esterase	This work
CE1	Fisuc_1568	FSU_2052	–	–	Yes	esterase	–	–
CE1	Fisuc_1569	FSU_2054	–	–	Yes	esterase	–	–
CE1	Fisuc_1768	FSU_2268			Yes	feruloyl esterase	–	–
CE1	Fisuc_1948	FSU_2468	–	–	Yes	esterase	–	–
CE1	Fisuc_2396	–	CBM4	–	Yes	esterase	–	–
CE2	Fisuc_1641	FSU_2130	–	–	Yes	esterase	esterase	This work
CE6	Fisuc_1766	FSU_2266	CBM6	BTD	Yes	acetyl xylan esterase	acetyl xylan esterase	[36]
CE6	Fisuc_1767	FSU_2267	CBM6	BTD	Yes	acetyl xylan esterase	acetyl xylan esterase	[36], [37]
CE6	Fisuc_2315	FSU_2864	–	–	Yes	esterase	–	–
CE6	Fisuc_2534	FSU_3103	CBM6	BTD	Yes	acetyl xylan esterase	xylan esterase	This work
CE6	Fisuc_2800	FSU_0054	–	–	Yes	acetyl xylan esterase	–	–
CE8	Fisuc_0679	FSU_1115	CBM35	–	No	pectin esterase	–	–
CE12	Fisuc_1995	FSU_2518	–	–	Yes	esterase	–	–
CE12	Fisuc_2478	FSU_3044	CBM35	–	Yes	esterase	–	–
CE12	Fisuc_2479	FSU_3045	CBM35	–	No	esterase	–	–
CE15	Fisuc_2348	FSU_2898	–	–	No	esterase	–	–

a FSU locus tags refer to the equivalent ORF call in the *F. succinogenes* genome sequence project described by GenBank accession: CP002158.

b Basic Terminal Domain (BTD) is also known as *F. succinogenes*-specific paralogous module 1 (FPm-1) [46].

### Non-catalytic CBM-containing proteins

The genome of *F. succinogenes* contains seven genes that appear to encode for CBM domains with no apparent catalytic function (Table 5). These include members of CBM4, CBM6, CBM30 and CBM51. Members of CBM4 and CBM30 domains typically bind to single chain cellulose, CBM6 to single chain hemicelluloses, and family CBM51 to galactose. The function of these proteins is unclear.

**Table 5:** Known genes predicted to encode only carbohydrate binding modules from the *Fibrobacter succinogenes* S85 genome.

CAZy Family	Fisuc Locus	FSU Locus^a^	Basic Terminal domain^b^	Signal Peptide	In Silico Prediction	Characterized Activity	Reference
CBM4	Fisuc_1931	FSU_2441	–	No	carbohydrate binding module	–	–
CBM6	Fisuc_1774	FSU_2273	–	Yes	carbohydrate binding module	–	–
CBM6	Fisuc_2485	FSU_3051	BTD	Yes	carbohydrate binding module	none found	This work
CBM30	Fisuc_1525	FSU_2007	–	Yes	carbohydrate binding module	–	–
CBM51	Fisuc_0215	FSU_0622	–	No	carbohydrate binding module	–	–
CBM51	Fisuc_0401	FSU_0816	–	No	carbohydrate binding module	–	–
CBM51	Fisuc_1656	FSU_2145	–	Yes	carbohydrate binding module	–	–

a FSU locus tags refer to the equivalent ORF call in the *F. succinogenes* genome sequence project described by GenBank accession: CP002158.

b Basic Terminal Domain (BTD) is also known as *F. succinogenes*-specific paralogous module 1 (FPm-1) [46].

### Non-CBM-containing adherence proteins

In addition to these non-catalytic CBM proteins, *F. succinogenes* is known to produce a number of cellulose-binding proteins localized to the cell surface that facilitates its adherence to crystalline cellulose [33], [50]. Previous proteomic analyses of *F. succinogenes* outer membrane proteins identified a cellulose-binding protein potentially involved in this process [50], and inhibition of this protein using antibodies significantly reduced the ability of *F. succinogenes* to bind to cellulose [50]. Structure-based analysis of this protein revealed a specific domain with strong homology to a domain in the slime mold *Dictyostelium discoideum*, and this protein was annotated as a fibro-slime domain-containing protein [33]. A total of 10 fibro-slime proteins were identified in that analysis, and we searched the *F. succinogenes* genome for evidence of the underlying genes encoding these proteins. We confirmed the presence of these 10 paralogs and mapped them to 10 different genes in the *F. succinogenes* genome (Fisuc_0377, Fisuc_1326, Fisuc_1327, Fisuc_1474, Fisuc_1475, Fisuc_1979, Fisuc_2031, Fisuc_2293, Fisuc_2471, Fisuc_2484). The large number of paralogous fibro-slime genes in the *F. succinogenes* genome suggests that they may play an important role in the ability of *F. succinogenes* to bind to cellulose.

An analysis of these genes on the *F. succinogenes* genome shows that they are typically surrounded by groups of hypothetical proteins with the exception of the gene encoding fibro-slime protein Fisuc_2484, which appears in a putative operon (Fisuc_2484-Fisuc_2487) containing a CBM6, a hypothetical protein, and an ABC-transporter related protein. Given that CBM6 domains bind to hemicelluloses, this fibro-slime protein may play a role in hemicellulose degradation. Finally, there are two clusters of fibro-slime proteins that have putative cellulases located near by. These include the clustered genes encoding fibro-slime proteins Fisuc_1474 and Fisuc_1475, which has a GH45 cellulase immediately upstream (Fisuc_1473) and the gene encoding fibro-slime protein Fisuc_2031, which has a GH9 cellulase (Fisuc_2033) located downstream. The close proximity of these genes to two cellulases may indicate a role in their activity.

Recently, type IV pilin proteins have been implicated in *F. succinogenes* adherence [33], as adherence deficient mutants lacked some of these proteins. A survey of the *F. succinogenes* genome reveals the presence of a small number of type IV pilin genes (Fisuc_0251-Fisuc_0253; Fisuc_0956; and Fisuc_1016-Fisuc_1017). Although a complete set of genes necessary to produce pili are not present in the genome, these pilin genes may be used by *F. succinogenes* to adhere to cellulose in a manner similar to how *Escherichia coli* and other gram negative bacteria adhere to solid substrates [51].

The reliance of *F. succinogenes* on membrane-bound adherence proteins may also be correlated to its protein secretion systems. *F. succingoenes* appears to contain the Sec-dependent and type II secretion pathways. In other bacteria such as the plant pathogen *Xanthomonas campestris*, the Sec-dependent and type II secretion systems are used to secrete hydrolytic enzymes like cellulases, chitinases, and pectate lyases to facilitate its pathogenic lifestyle [52]. Type II secretion pathways have also been implicated in the adherence of human pathogenic bacterial cells to the host epithelium [52], and given that *F. succinogenes* requires adherence to cellulose for efficient hydrolysis, this pathway may play a role in cell adherence.

### Utilization of cellulose hydrolysis products

Cellodextrins liberated by the cellulolytic action of *F. succinogenes* can be transported into the cell for further processing via a cellodextrin-specific transporter. For example, the *F. succinogenes* genome encodes for extracellular-solute binding proteins that are known to participate in the active transport of solutes through the ABC active transport mechanism. These proteins, which are found attached to the cell surface and help recognize and bind specific solutes, could be used for cellodextrin transport. This type of transporter specificity is known in other cellulolytic bacteria including *C. thermocellum* [53], and an alignment of the known cellodextrin-specific extracellular-solute binding protein from this bacterium shows strong homology with a extracellular-solute binding protein (Fisuc_0617) in *F. succinogenes* (data not shown) [10].

Once incorporated into the cell, cellodextrins can be sequentially converted to glucose-1-phosphate by the action of cellodextrin phosphorlyase, whose presence has been demonstrated in cell-free extracts. The *F. succinogenes* genome appears to contain only a single cellobiose phosphorylase gene (Fisuc_2900), and physiological studies suggest that this enzyme can reversibly catalyze both degradation and synthesis of cellodextrins. This differs from the case of some cellulolytic bacteria such as *C. thermocellum*, in which cellobiose is metabolized via one phosphorlylase and longer cellodextrins are metabolized by a second phosphorylase [54]–[56], but cellodextrins are not produced in significant quantities by either phosphorylase. An alternative pathway would be degradation of the cellodextrins in either the cell wall or periplasmic space by the chloride-stimulated cellobiohydrolases (CLCBA, Fisuc_2992) known to be required for degradation of crystalline cellulose [57]. Cellobiose would then be transported via a similar system to that proposed for cellodextrins.

**Figure 2:**
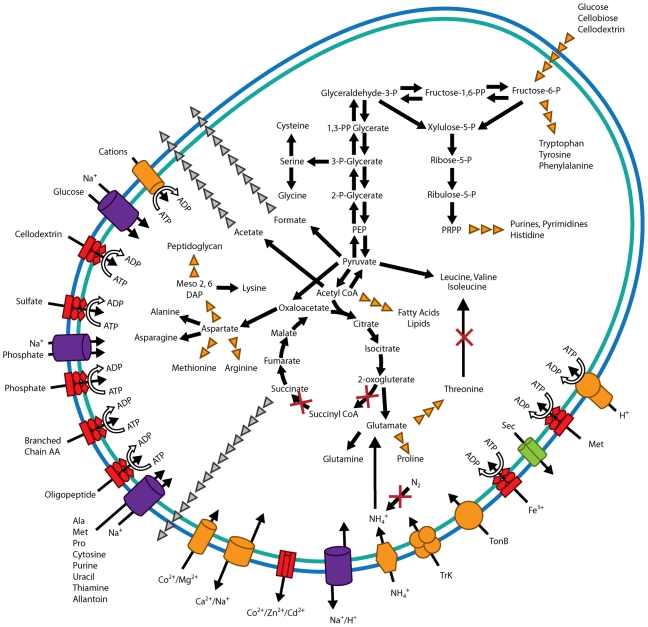
An overview of metabolism and transport in *Fibrobacte succinogenes* S85. Enzymes missing from metabolic pathways are indicated with a red cross. The major fermentative products succinate, acetate, and formate are shown with gray arrows indicating their export out of the cell. Predicted transporters are also shown, including sodium ion channel protein transporters in purple, ABC transporters in red, sec-dependent protein export in green, and other substrates in blue. Export or import of solutes is shown through the direction of the arrow through the transporter. Energy coupling mechanisms are also shown, including solutes transported by channel proteins; secondary transporters with two arrows into the cell indicating the solute and coupling ion; ATP-driven transporters with an ATP hydrolysis reaction; and transporters with an unknown energy-coupling mechanism, shown with a single arrow. Some multi-step pathways are not fully-represented, and are denoted with orange arrowheads. Abbreviations: AA, amino acids; Ala, alanine; Met, methionine; Pro, proline; PRPP, 5*′*-phospho-α-D-ribose 1-diphosphate; PEP, phosphoenolpyruvate; Meso 2,6 DAP, meso-2,6- diaminopimelic acid.

The catabolism of cellulose hydrolysis products by *F. succinogenes* was characterized further by performing a metabolic reconstruction analysis using the software program PRIAM [58] to generate KEGG pathway maps [59]. *F. succinogenes* is a strict anaerobe that produces a mixture of succinate, acetate and formate as fermentation end products [3], [7]. The metabolic reconstruction shows this strain contains both a complete Embden-Meyerhof-Parnas pathway and an incomplete citric acid cycle, lacking both an α-ketoglutarate dehydrogenase and a succinyl-CoA-synthase (Figure 2). This agrees with enzymatic evidence [60] that *F. succinogenes* utilizes its incomplete citric acid cycle for the production of succinate, the bacterium's major fermentative end product. Specifically, the reconstruction shows that *F. succinogenes* contains both a phosphoenolpyruvate carboxykinase (Fisuc_2949) and a pyruvate carboxylase (Fisuc_0845) that can reversibly convert PEP and pyruvate, respectively, to oxaloacetate, which is sequentially converted to malate, fumarate, and succinate. The reconstruction also suggests that energy production is likely facilitated through the transfer of electrons from a carrier such as menaquinone to fumarate through the action of a membrane-bound fumarate reductase (Fisuc_2493 and Fisuc_2494), resulting in the production of succinate. Previously, it was reported that fumarate reduction can be coupled to hydrogen oxidation in *F. succinogenes* [61]. However, a search of the *F. succinogenes* genome for the presence of a hydrogenase using over 700 previously identified enzymes [62], did not find any evidence for a hydrogenase in *F. succinogenes*. *F. succinogenes* can also produce formate and acetyl-CoA through the action of formate C-acetyltransferase (Fisuc_1044) with the assistance of a pyruvate-formate lyase activating enzyme (Fisuc_1047).

The metabolic reconstruction predicts that *F. succinogenes* does not have an Entner-Doudoroff pathway and that the glyoxylate shunt is also absent. This bacterium also appears to have incomplete pathways for the utilization of galactose, mannose, fructose and pentose sugars. These predictions are in agreement with our finding that *F. succinogenes* is unable to grow on any saccharides other than cellulose and its soluble components (glucose, cellobiose, and cellodextrins) (Table 1).

### Comparison of polysaccharide-degrading strategies with those of other ruminal bacteria

In addition to *F. succinogenes*, several other well-known polysaccharide-degrading bacteria have been isolated from the rumen including *Butyrivibrio proteoclasticus* B316 [63], *Prevotella ruminicola* 23 [64] and *Ruminococcus flavefaciens* FD-1 [65]. These species are thought to work synergistically with *F. succinogenes* to degrade plant biomass in the rumen, and utilize different strategies to deconstruct polysaccharides [2]. To gain an understanding of these systems we compared the carbohydrate-degrading potential of these bacteria as shown in Table S3. In general, these bacteria have an apparent specificity in their carbohydrate-active enzymes with some overlap. *F. succinogenes* and *R. flavefaciens* FD-1 are known to be prolific cellulose-degrading specialists, whereas *P. ruminicola* 23 is a generalist capable of degrading and utilizing many different polysaccharides, but not cellulose [64]. *B. proteoclasticus* B316 also cannot degrade cellulose, but can degrade and utilize xylan, starch, and pectin [63]. These capabilities are reflected in their carbohydrate-active enzyme profiles.

Both *F. succinogenes* S85 and *R. flavefaciens* FD-1 contain a number of cellulases from the same families including members of the GH5 and GH9. *F. succinogenes* also contains a number of enzymes in the GH8, GH45, and GH51 families classified as cellulases that are not found in *R. flavefaciens*, whereas a GH48 exoglucanase is present in *R. flavefaciens* FD-1 but not in *F. succinogenes*. The higher diversity of cellulases in *F. succinogenes*, relative to *R. flavefaciens* FD-1 may account for its ability to degrade all known allomorphs of cellulose, including the highly stable, chemically regenerated cellulose II. However, the relatively weak hydrolytic capacity of these enzymes to degrade crystalline cellulose *in vitro* and the *in vivo* requirement for direct adherence to cellulose, as discussed above, indicates that *F. succinogenes* must use other mechanisms such as adherence molecules or “atypical” cellulases such as the GH9s, which are thought to synergistically work with other cellulases [29].

*B. proteoclasticus* B316 and P. *ruminicola* 23 also have a number of enzymes within these GH families, but in much smaller numbers than are found in *F. succinogenes* and *R. flavefaciens* FD-1. These GH family enzymes appear to be xylanases and other hemicellulases, which corresponds to the reported inability of the former two organisms to degrade cellulose. These are complemented by other *endo*- and *exo*-hemicellulases in the families GH2, GH3, GH10, GH16, GH43, and GH53, with the largest numbers of enzymes within the GH43s; each bacterium is predicted to have at least 10 copies of GH43. In addition, *B. proteoclasticus* B316 and *P. ruminicola* 23 contain xylanases in the family GH11 and GH44 that are not found in either *F. succinogenes* or *R. flavefaciens* FD-1. The large diversity of hemicellulases within these 4 bacteria corresponds well to their known polysaccharide-degrading and utilization mechanisms, although *F. succinogenes* is the only one that degrades xylan without using its hydrolytic products.

Finally, *B. proteoclasticus* B316 and *P ruminicola* 23 contain enzymes in the GH28, GH29, GH32, GH35, and GH38 families that are not found in either *F. succinogenes* or *R. flavefaciens* FD-1. These GHs include pectinases, fucosidases, fructanases, and mannosidases, which correspond to their more generalist lifestyle of degrading and utilizing a wider variety of polysaccharides.

In addition to the GHs, the CBMs found in these four ruminal bacteria also support their polysaccharide degrading strategies. For example, *F. succinogenes* contains members of CBM11, CBM30, and CBM51 whereas *B. proteoclasticus* B316, *P. ruminicola* 23, *R. flavefaciens* FD-1 do not. Both CBM11 and CBM30 are known to bind to cellulose in *F. succinogenes* [31], providing further evidence for its ability to degrade cellulose. The only shared cellulose-binding CBMs between *F. succinogenes* and *R. flavefaciens* FD-1 is CBM4, which is known to bind to single chain cellulose. Furthermore, *F. succinogenes* has 20 copies of CBM6, more than 4-fold greater than any of the other three ruminal bacteria. These domains, which are found associated with hemicellulases in *F. succinogenes*, may indicate that they are the preferred modules for facilitating hemicellulose deconstruction. *R. flavefaciens* FD-1 and *B. proteoclasticus* B316 may also utilize organism-specific CBMs for hemicellulose and cellulose degradation, as CBM13 and nine copies of CBM2 are found in these bacteria, respectively. The apparent specificity of these CBMs in their respective organisms may indicate different strategies for binding to hemicelluloses such as xylan, or perhaps may reflect different preferences for different plant tissue types. *P. ruminicola* does not appear to have any organism-specific CBMs, when compared to the three other ruminal bacteria, but utilizes a variety of CBMs reflecting its more general polysaccharide-degrading lifestyle.

## Discussion

Here we report for the first time, the complete genome sequence for the cellulolytic ruminal bacterium *Fibrobacter succinogenes*. This also represents the first complete genome of a bacterium belonging to the phylum Fibrobacteres. The rumen is an environment tailored for the conversion of plant biomass into volatile fatty acids usable by its host and it is apparent that ruminal microbes like *F. succinogenes* are specialized for this process. *F. succinogenes*, as its name implies, is a producer of succinate, and our metabolic reconstruction analysis confirms that this, along with acetate and formate, are major fermentative end products.

However, unlike other ruminal bacteria that derive their energy from many different polysaccharide sources, *F. succinogenes* is specialized for using only cellulose. Our physiological assays and analysis of the *F. succinogenes* carbohydrate-degrading machinery reveals this property, and further suggests a specific model of plant polysaccharide deconstruction. The *F. succinogenes* genome encodes for a number of enzymes capable of degrading a wide array of polysaccharides and it is likely that it uses these to remove carbohydrates like xylan in order to gain access to cellulose. This is supported by our finding that while *F. succinogenes* can hydrolyze these substrates, it can not metabolize the end products as carbon sources. For example, *F. succinogenes* can hydrolyze xylan into xylose, but can not utilize this as an energy source because it lacks both a xylose permease for transport into the cell and a xylose isomerase.

The polysaccharide degrading strategy of *F. succinogenes* is markedly different from other cellulolytic bacteria, not only in its specialization on cellulose, but also in its method of cellulose degradation. Adherence of the bacterium to solid cellulosic substrates appears to be a requirement for the degradation of cellulose [13], [14], and little cellulase activity is detected in the culture medium [40]. *F. succinogenes* is one of only a few organisms reported to rapidly degrade all allomorphs of cellulose, including cellulose II [66]. Previous work has shown that the organism does not possess extensive secreted cellulases or cellulosomal structures like other ruminal organisms such as *Ruminococcus flavefaciens* (reviewed in [56]). Identified cellulolytic enzymes show low homology to cellulases from other organisms [29] and the *F. succinogenes* cellulases that have been cloned to date show poor performance on crystalline cellulose both alone and in combinations [29], [67]. The lack of identifiable crystalline cellulose-binding domains and the poor performance of the identified enzymes on crystalline cellulose – despite active degradation of this substrate by whole cells – has led to the hypothesis that cellulose degradation by this species relies on an unusual degree of cell-enzyme synergy [56], and perhaps even utilizes a cell-based, non-enzymatic process [22]. A number of benefits appear to accrue from the novel lifestyle of *F. succinogenes* [68], but it is not yet clear how the genes identified in the organism contribute to its success. It is likely that the unconventional mode of cellulose degradation by *F. succinogenes* accrues at least in part from an unusual combination of cellulases distributed into certain families which are relatively poorly represented among microbes that employ more conventional modes of cellulose degradation.

### Models of polysaccharide degradation for *F. succinogenes*

Several models can be proposed for cellulose degradation by *F. succinogenes.* Tenable models must be in accord with observations that *F. succinogenes:* i) produces trace amounts of measurable soluble cellulase activity in fermentations actively degrading cellulose [7]; ii) has an absolute requirement for adherence to effect cellulose degradation [13], [14]; iii) is unable to degrade cellulose as cell-free extracts [40]; and iv) can not bind to or degrade crystalline cellulose when certain non-cellulase genes are mutated [33]. Arguably, the two most studied systems for cellulose degradation are the soluble enzyme systems of *Trichoderma reesei* and the cellulosomal systems of bacteria like *C. thermocellum.* Both models can be eliminated for *F. succinogenes* because it does not produce soluble cellulases and our genomic analysis reveals no homologs to known cellulosomal proteins. Therefore, these models do not successfully meet all of the criteria required for *F. succinogenes* cellulose degradation, and we consider other possible models here.

One proposed model describes the growth of *F. succinogenes* as a biofilm on the cellulose surface, similar to the case of ruminococci [69]. *F. succinogenes* is thought to rely on fibro-slime proteins to attach to its substrate and initiate or support cellulose deconstruction. Proteins such as the fibro-slime [33], [50] and type IV pilin structures [70] would facilitate cell-surface attachment to the substrate and mediate close contact of both GH and CBM-containing enzymes to polysaccharides. Other unidentified modules in the *F. succinogenes* genome may be expected to play similar roles to CBMs or the dockerins and scaffoldins that facilitate cellulosomal degradation in ruminal bacteria like *Ruminococcus*. The specific mechanism of substrate degradation has been suggested to proceed through the localization of cellulases and hemicellulases on the cell membrane. This may indeed be the case for hemicellulases, many of which contain the *F. succinogenes*-specific paralogous module 1. However, sequence analysis indicates that none of the cellulases possess this domain or any other previously-reported anchoring domain, making it unlike that they are attached to the membrane. This localization is also not supported by results from isolation of individual membrane fractions of the organism [71].

*F. succinogenes* also appears to employ 'atypical' cellulases that may obviate the need for extensive CBMs [29]. In particular, a GH9 cellulase has been characterized that synergistically increases the hydrolytic ability of other cellulases like Cel51 and Cel8B [27], [29]. The large number of GH9s in the genome, and their potential localization to the cell membrane (Table 2) could act to increase the efficiency of many of *F. succinogenes* cellulases. The combination of adherence molecules, carbohydrate-binding molecules, and interacting cellulases may display a hydrolytic synergy that mediates efficient cellulose degradation as has been previously suggested [70], [72]. However, it should be noted that cloned, expressed, purified and characterized *F. succinogenes* cellulases cannot significantly degrade crystalline cellulose, either alone or in combination, making it difficult to understand how they might function to quickly and effectively depolymerize all the allomorphs of cellulose.

In addition, two other cellulolytic mechanisms have been suggested. *Cytophaga hutchinsonii*, a bacterium within the *Cytophaga-Flavobacterium-Bacteroides* phylum that is phylogenetically-related to *F. succinogenes*, is known to associate tightly with cellulose. This bacterium has been proposed to degrade cellulose by disrupting cellulose fibers and taking up individual cellulose chains through the outer membrane [73]. Upon reaching the periplasmic space, these chains would be cleaved by endoglucanases. This presents an intriguing model for *F. succinogenes*, which could thus gain direct access to the hydrolytic products of cellulose (glucose, cellobiose, and cellodextrins). However, *F. succinogenes* and *C. hutchinsonii* share few cellulase homologs and this model of cellulose degradation by *C. hutchinsonii* is thought to be facilitated by its ability to move in parallel across cellulose fibers using gliding motility [74], during which cellulose chains could be stripped from fibers as it glides across its substrate. In contrast, motility in *F. succinogenes* has not been demonstrated, nor did we find any known motility genes in its genome. If *F. succinogenes* were to employ an approach similar to *C. hutchinsonii*, it may be accomplished using the previously described fibro-slime proteins. In the slime mold *Dictyostelium discoideum*, slugs produce a cellulose sheath that enables it to move. The related fibro-slime genes in *F. succinogenes* may play a similar role, enabling it to move in an analogous manner without apparent motility in solution. In this regard, it is interesting that degradation of crystalline cellulose by *F. succinogenes* appears to occur along the crystallographic axis, suggesting a directionality of the hydrolytic process [13], [14].

Alternatively, *F. succinogenes* may follow a model proposed for the α-proteobacterium *Sphingomonas* sp. A1 [75]. This bacterium appears to form “pits” across its cell membrane that act as channels that can import and depolymerize macromolecules like alginate [76]. Import of macromolecules through the membrane is thought to occur through two high-affinity periplasmic proteins facilitated by an ABC transporter. The degradation of macromolecules would occur in the cytoplasm. Comparison of the genes implicated in *Sphingomonas* sp. A1 pit formation with the *F. succinogenes* genome reveals a number of homologs including type IV pilin molecules and ABC transporters (data not shown). However, electron microscopic analysis of *F. succinogenes* [13] does not reveal the presence of pits as has been shown for *Sphingomonas* sp. A1. While it is possible that these pits may be too small to be detected using electron microscopy, one consideration is that alginate and cellulose are very different molecules with respect to their higher order structure, and this model would require the disassembly (decrystallization and delamination) of individual cellulose fibers or microfibrils before they could be imported through these pits.

### Conclusion

The mechanism by which *F. succinogenes* degrades cellulose is not obvious and stands in stark contrast to the strategies used by other cellulolytic microbes. The availability of the *F. succinogenes* genome will serve to increase our understanding of its unique cellulose degrading properties and provide insight into the peculiar biology of this bacterium and its phylum, given the association of different *Fibrobacter* species in ruminants and other animals [4], [77], [78]. Furthermore, with the increasing number of ruminal bacterial genomes becoming available, we will be able to leverage this data to begin understanding how these microbes interact within the rumen and their impact on ruminant health and animal performance. Finally, from a biotechnological perspective, understanding how *F. succinogenes* accomplishes polysaccharide hydrolysis will help inform our own efforts to optimally convert cellulosic material for the production of biofuels.

## Materials and Methods

### DNA extraction, genome sequencing and finishing

The type strain *Fibrobacter succinogenes* S85 (ATCC 19169^T^) was obtained from Dr. Cecil Forsberg (University of Guelph). We grew cultures of *F. succinogenes* in a modified Dehority medium [14] supplemented with 4 g cellulose/L for 48 h at 39 °C. Genomic DNA was then prepared as described by Stevenson and Weimer [5].

The genome of *F. succinogenes* S85 was sequenced at the DOE Joint Genome Institute (JGI) using a combination of Illumina [79] and 454 technologies [80]. An Illumina GAii shotgun library with reads of 477 Mb, a 454 Titanium draft library with average read length of 243 bases, and a paired end 454 library with average insert size of 20.5 Kb were generated for this genome. All general aspects of library construction and sequencing performed at the JGI can be found at http://www.jgi.doe.gov/. Illumina sequencing data was assembled with VELVET [81], and the consensus sequences were shredded into 1.5 kb overlapped fake reads and assembled together with the 454 data. Draft assemblies were based on 343 Mb 454 draft data, and 454 paired end data. Newbler assembly parameters are -consed -a 50 -l 350 -g -m -ml 20.

The initial assembly contained 19 contigs in 1 scaffold. We converted the initial 454 assembly into a Phrap assembly by making fake reads from the consensus, collecting the read pairs in the 454 paired end library. The Phred/Phrap/Consed software package (http://www.phrap.com) was used for sequence assembly and quality assessment [82]–[84] in the following finishing process. After the shotgun stage, reads were assembled with parallel Phrap (High Performance Software, LLC). Possible mis-assemblies were corrected with the gapResolution software (Cliff Han, unpublished), Dupfinisher [85], or sequencing cloned bridging PCR fragments with subcloning or transposon bombing (Epicentre Biotechnologies, Madison, WI). Gaps between contigs were closed by editing in Consed, by PCR and by Bubble PCR primer walks (J.-F. Cheng, unpublished and described at http://www.jgi.doe.gov). A total of 103 additional reactions were necessary to close gaps and to raise the quality of the finished sequence. The completed genome sequence of *F. succinogenes* S85 is 3,842,636 bases, with an error rate less than 1 in 100,000 bp. The *F. succinogenes* S85 genome and annotation can be obtained through GenBank under accession CP001792.1.

### Genome annotation

The sequence of *F. succinogenes* was annotated at Oak Ridge National Laboratory (ORNL) using their genome annotation pipeline. This includes the application of a number of annotation programs beginning with open reading frame (ORF) prediction using Prodigal [86] followed by manual annotation using the JGI GenePrimp pipeline [87]. Automated protein function prediction was then performed using a number of databases including protein domains (Pfam) [88], UniProt [89], TIGRFAMs [90], KEGG [59], Interpro [91], and COG [20]; metabolic reconstruction analysis using PRIAM [58]; signal peptide prediction using SignalP [39]; tRNA prediction using tRNAscan-SE [92]; and rRNA prediction using RNAmmer [93]. These annotations can be publicly accessed at http://genome.ornl.gov/microbial/fs85/. The ORNL-generated annotation predicts 3,087 ORFs; however the final GenBank submitted annotation contains only 3,085 ORFs, which we report here. This difference is due to the GenBank standard submission process which removed two predicted ORFs considered to be spurious.

### Whole-genome analysis

A protein comparison analysis was performed for the *F. succinogenes* proteome by using BLAST [94] to query against a local database composed of proteins from 1,172 microbial genomes (http://www.ncbi.nlm.nih.gov/genomes/lproks.cgi?view=1, accessed: 06/17/2010). Those proteins that had a BLAST hit (e-value: 1e-05) were recorded and divided into two different categories: proteins that had a putative assigned function, and those that matched to hypothetical proteins. Proteins that did not have a significant BLAST hit were designated as species- or genus-specific proteins. These data were then used to generate a taxonomic distribution analysis as shown in Figure 1. This was done by counting the phyla/group membership for the top hit of each protein in the *F. succinogenes* proteome against the local database of microbial proteomes and expressing this as a percentage of the total number of *F. succinogenes* proteins that could be assigned a closest taxonomic member.

A clusters of orthologous groups (COG) [20] analysis was performed using the *F. succinogenes* genome as follows. Predicted proteins from *F. succinogenes* were queried against a COG database retrieved from NCBI (ftp://ftp.ncbi.nih.gov/pub/mmdb/cdd/, accessed: 06/19/2010) using RPSBLAST [95] (e-value 1e-05). The top RPBLAST hit for each protein was tabulated and placed into each COGs respective category as shown in Table 2.

We also analyzed the predicted carbohydrate-active enzymes (CAZymes) [21] for *F. succinogenes* available through the CAZy database (http://www.cazy.org/, accessed: 03/01/2011). However, because there are many legacy enzymes in this database that have not been correlated to the current annotation of the genome, we used a custom Perl script to tabulate a single set of CAZymes that corresponds to the genome annotation as shown in Table 2.

### Polysaccharide hydrolysis and growth measurements

Experiments were conducted under a CO_2_ gas phase in triplicate 60 mL glass serum vials that contained 10 mL of modified Dehority medium [96] supplemented with the indicated polysaccharide. Cultures were incubated without shaking at 39 ^°^C for 72 h. Hydrolysis of polysaccharides was measured as release of reducing sugars by the dinitrosalicylic acid method [97], using glucose as standard. Growth on polysaccharides was not measured directly, but was instead measured as succinate production [98], using HPLC [96].

### Enzyme cloning, expression, and characterization

Genes were cloned, amplified, and expressed as previously described [22] . Briefly, the presence of signal sequences within target genes was determined using the SignalP software [39]. Primers were then selected (Table S2) and these genes were amplified without signal sequences from DNA extracted from *F. succinogenes*. The resulting amplicons were cloned, expressed and characterized as described previously [22]. The range of substrates used for evaluation was expanded to include the following synthetic substrates: AZCL-Arabinan, AZCL-Arabinoxylan, AZCL-beta-Glucan, AZCL-Galactomannan, AZCL-HE Cellulose, AZCL-Xyloglucan, 4-methylumbelliferyl-β-D-cellobioside (MUC), 4-methylumbelliferyl-β-D-xylopyranoside (MUX), and 4-methylumbelliferyl-β-D-glucoyranoside (MUG), pNP-β-glucoside, pNP-β-cellobioside, 4-methylumbelliferyl-α-D-arabinofuranoside (MUA), 4-methylumbelliferyl-β-D-lactopyranoside (MUL), 5-Bromo-4-chloro-3-indolyl α-D-galactopyranoside (X-α-Gal, XAG), and 5-Bromo-4-chloro-3-indolyl β-D-galactopyranoside (X-gal, XG). A total of 14 new GH family members, 3 new CE family members, 1 PL family member, and 1 CBM family member with no identified enzymatic activity investigated in this manner, as shown in Table S2. The observed activities of the cloned proteins are shown in Table S4.

## Supporting Information

Table S1 Clusters of orthologous group (COG) analysis for *Fibrobacter succinogenes* S85.
(DOC)

Table S2 Carbohydrate-active enzymes encoded by the *Fibrobacter succinogenes* S85 genome.
(DOC)

Table S3 Comparison of carbohydrate-degrading enzymes (CAZymes) encoded by the genomes of the 4 ruminant bacteria *Fibrobacter succinogenes* S85 (Fsuc), *Butryvibrio proteoclasticus* B316 (Bpro), *Prevotella ruminocola* 23 (Prum), and *Ruminococcus flavefaciens* FD-1 (Rfla).
(DOC)

Table S4 Primers used to clone selected CAZymes in *F. succinogenes* and their tested polysaccharide activity.
(DOC)

Text S1 Glycogen biosynthesis and utilization.
(DOC)

Text S2 Amino acid synthesis and nitrogen assimilation.
(DOC)

Text S3 Fatty acid synthesis and catabolism.
(DOC)

Text S4 Vitamin biosynthesis.
(DOC)

Text S5 Transporters.
(DOC)

Text S6 Antibiotic production and resistance.
(DOC)

Text S7 DNA Repair.
(DOC)

Text S8 CRISPRs, insertion sequences, and genomic islands.
(DOC)
