# Supplementary material for: The Complete Genome Sequence of Fibrobacter succinogenes S85 Reveals a Cellulolytic and Metabolic Specialist
Source: PLoS One. 2011 Apr 19;6(4):e18814. doi: 10.1371/journal.pone.0018814 (PMC3079729; doi:10.1371/journal.pone.0018814)
Supplement: Table S1 — Clusters of orthologous group (COG) analysis for Fibrobacter succinogenes S85. (DOC) [file pone.0018814.s001.doc]

**Table S1**. Clusters of orthologous group (COG) analysis for *Fibrobacter succinogenes* S85.

| **COG Category** | | **Code** | **Totals** |
| --- | --- | --- | --- |
| **Information storage and processing** | |
|  | Translation, ribosomal structure and biogenesis | J | 152 (7.84%) |
|  | RNA processing and modification | A | 1 (0.05%) |
|  | Transcription | K | 100 (5.16%) |
|  | Replication, recombination and repair | L | 120 (6.19%) |
|  | Chromatin structure and dynamics | B | 0 (0%) |
| **Cellular processes and signaling** | | | |
|  | Cell cycle control, cell division, chromosome partitioning | D | 25 (1.29%) |
|  | Nuclear structure | Y | 0 (0%) |
|  | Defense mechanisms | V | 36 (1.86%) |
|  | Signal transduction mechanisms | T | 97 (5.01%) |
|  | Cell wall/membrane/envelope biogenesis | M | 206 (10.63%) |
|  | Cell motility | N | 19 (0.98%) |
|  | Cytoskeleton | Z | 0 (0%) |
|  | Extracellular structures | W | 0 (0%) |
|  | Intracellular trafficking, secretion, and vesicular transport | U | 52 (2.68%) |
|  | Posttranslational modification, protein turnover, chaperones | O | 68 (3.51%) |
| **Metabolism** | | | |
|  | Energy production and conversion | C | 92 (4.75%) |
|  | Carbohydrate transport and metabolism | G | 138 (7.12%) |
|  | Amino acid transport and metabolism | E | 165 (8.51%) |
|  | Nucleotide transport and metabolism | F | 60 (3.1%) |
|  | Coenzyme transport and metabolism | H | 95 (4.9%) |
|  | Lipid transport and metabolism | I | 56 (2.89%) |
|  | Inorganic ion transport and metabolism | P | 68 (3.51%) |
|  | Secondary metabolites biosynthesis, transport and catabolism | Q | 31 (1.6%) |
| **Poorly characterized** | | | |
|  | General function prediction only | R | 233 (12.02%) |
|  | Function unknown | S | 124 (6.4%) |
| **Total ORFs COG Annotated** | | | **1,938** |
