# Supplementary material for: The Complete Genome Sequence of Fibrobacter succinogenes S85 Reveals a Cellulolytic and Metabolic Specialist
Source: PLoS One. 2011 Apr 19;6(4):e18814. doi: 10.1371/journal.pone.0018814 (PMC3079729; doi:10.1371/journal.pone.0018814)
Supplement: Table S2 — Carbohydrate-active enzymes encoded by the Fibrobacter succinogenes S85 genome. (DOC) [file pone.0018814.s002.doc]

**Table S2.** Carbohydrate-active enzymes encoded by the *Fibrobacter succinogenes* S85 genome.

| **CAZy**  **Family**^a^ | **Fisuc**  **Locus** | **FSU Locus**^b^ | **CBM Family** | **Basic Terminal domain**^c^ | **Signal Peptide** | **In Silico Prediction** | **Characterized Activity** | **Reference** |
| --- | --- | --- | --- | --- | --- | --- | --- | --- |
| **Glycoside Hydrolases** | | | | | | | | |
| GH2 | Fisuc_1788 | FSU_2288 | – | BTD | Yes | β-galactosidase | – | – |
| GH2 | Fisuc_3049 | FSU_0315 | – | – | No | β-galactosidase | β-galactosidase | This work |
| GH3 | Fisuc_1751 | FSU_2249 | – | – | Yes | β-xylosidase | – | – |
| GH3 | Fisuc_1985 | FSU_2508 | – | – | Yes | β-xylosidase | – | – |
| GH3 | Fisuc_2065 | FSU_2592 | – | – | Yes | β-xylosidase | cellobiosidase | This work |
| GH5 | Fisuc_0728 | FSU_1166 | – | – | Yes | mannanase | β-mannanase | This work |
| GH5 | Fisuc_0786 | FSU_1228 | – | – | Yes | cellulase | cellulase | This work |
| GH5 | Fisuc_0897 | FSU_1346 | – | – | Yes | cellulase | cellulase |  |
| GH5 | Fisuc_1224 | FSU_1685 | – | – | Yes | cellulase | cellulase | This work |
| GH5 | Fisuc_1523 | FSU_2005 | – | – | Yes | cellulase | cellulase | This work |
| GH5 | Fisuc_1584 | FSU_2070 | – | – | No | cellulase | – |  |
| GH5 | Fisuc_1661 | FSU_2150 | – | – | Yes | endoglucanase | – | – |
| GH5 | Fisuc_2011 | FSU_2534 | – | – | Yes | cellulase | – | – |
| GH5 | Fisuc_2230 | FSU_2772 | – | – | Yes | endoglucanase | cellulase |  |
| GH5 | Fisuc_2364 | FSU_2914 | – | – | Yes | cellulase | cellulase |  |
| GH5 | Fisuc_2933 | FSU_0196 | CBM4 | – | No | mannanase | β-mannanase | This work |
| GH5 | Fisuc_3081 | FSU_0347 | – | – | Yes | cellulase | cellulase | This work |
| GH8 | Fisuc_0207 | FSU_0613 | – | – | No | Endoglucanase | xylanase |  |
| GH8 | Fisuc_0241 | FSU_0651 | – | – | No | Endoglucanase | cellulase | This work |
| GH8 | Fisuc_0471 | FSU_0889 | – | – | Yes | endoglucanase | xylanase |  |
| GH8 | Fisuc_1219 | FSU_1680 | – | – | Yes | Endoglucanase | – | – |
| GH8 | Fisuc_1802 | FSU_2303 | – | – | No | endoglucanase | cellulase |  |
| GH8 | Fisuc_2579 | FSU_3149 | – | – | Yes | β-glucanase | cellulase |  |
| GH9 | Fisuc_0057 | FSU_0451 | – | – | No | β-glucanase | cellulase |  |
| GH9 | Fisuc_0393 | FSU_0809 | – | – | No | cellulase | – | – |
| GH9 | Fisuc_0394 | FSU_0810 | – | – | No | cellulase | – | – |
| GH9 | Fisuc_1531 | FSU_2013 | – | – | Yes | cellulase | – | – |
| GH9 | Fisuc_1859 | FSU_2361 | – | – | Yes | β-glucanase | cellulase |  |
| GH9 | Fisuc_1860 | FSU_2362 | – | – | Yes | cellulase | – |  |
| GH9 | Fisuc_2033 | FSU_2558 | – | – | Yes | endoglucanase | cellulase |  |
| GH9 | Fisuc_2362 | FSU_2912 | – | – | Yes | cellulase | cellulase |  |
| GH9 | Fisuc_2876 | FSU_0134 | – | – | No | cellulase | – | – |
| GH10 | Fisuc_0754 | FSU_1192 | – | – | Yes | xylanase | – | – |
| GH10 | Fisuc_0757 | FSU_1195 | – | – | Yes | xylanase | – | – |
| GH10 | Fisuc_1791 | FSU_2292 | CBM6 | BTD | Yes | xylanase | – |  |
| GH10 | Fisuc_1793 | FSU_2293 | CBM6 | BTD | yes | xylanase | xylanase |  |
| GH10 | Fisuc_1794 | FSU_2294 | CBM6 | BTD | Yes | xylanase | xylanase |  |
| GH10 | Fisuc_2303 | FSU_2851 | – | BTD | Yes | endoglucanase | – | – |
| GH10 | Fisuc_2992 | FSU_0257 | – | – | Yes | cellulase | – |  |
| GH11 | Fisuc_0362 | FSU_0777 | – | BTD | Yes | xylanase | xylanase |  |
| GH11 | Fisuc_2201 | FSU_2741 | – | BTD | Yes | xylanase | xylanase |  |
| GH11 | Fisuc_2442 | FSU_3006 | – | – | Yes | xylanase | xylanase |  |
| GH13 | Fisuc_0859 | FSU_1303 | – | – | No | α-amylase | – | – |
| GH13 | Fisuc_1932 | FSU_2442 | – | – | No | α amylase | – |  |
| GH13 | Fisuc_3103 | FSU_0369 | CBM48 | – | No | 1,4-α-glucan bra–hing enzyme | – | – |
| GH16 | Fisuc_1043 | FSU_1493 | – | – | Yes | β-glucanase | – | – |
| GH16 | Fisuc_2093 | FSU_2622 | – | – | No | β-glucanase | – | – |
| GH16 | Fisuc_2424 | FSU_2986 | – | BTD | Yes | endoglucanase | – | – |
| GH16 | Fisuc_2961 | FSU_0226 | – | – | Yes | β-glucanase | licheninase |  |
| GH18 | Fisuc_1530 | FSU_2012 | – | – | Yes | chitinase | cellulose binding |  |
| GH18 | Fisuc_2465 | FSU_3030 | – | – | Yes | chitinase | – | – |
| GH23 | Fisuc_0665 | – | – | – | Yes | 4-α-glucanotransferase | – | – |
| GH23 | Fisuc_1000 | FSU_1446 | – | – | No | lytic transglycosylase | – | – |
| GH23 | Fisuc_2988 | FSU_0253 | CBM50 | – | No | membrane lytic murein transglycosylase D | – | – |
| GH26 | Fisuc_0727 | FSU_1165 | CBM35 | – | Yes | mannanase | β-mannanase | This work |
| GH26 | Fisuc_0729 | FSU_1167 | CBM35 | – | Yes | mannanase | β-mannanase | This work |
| GH26 | Fisuc_0730 | FSU_1168 | – | – | Yes | mannanase | β-mannanase |  |
| GH26 | Fisuc_1266 | FSU_1729 | – | – | Yes | mannanase | – | – |
| GH26 | Fisuc_1688 | FSU_2181 | CBM35 | – | Yes | mannanase | – |  |
| GH27 | Fisuc_1773 | FSU_2272 | CBM6 | BTD | Yes | α-galactosidase | α-galactosidase |  |
| GH30 | Fisuc_1765 | FSU_2265 | CBM6 | BTD | No | xylanase | xylanase |  |
| GH30 | Fisuc_2919 | FSU_0182 | – | – | Yes | O-glycosyl hydrolase-like protein | – | – |
| GH30 | Fisuc_2943 | FSU_0206 | – | – | Yes | xylanase | – | – |
| GH39 | Fisuc_0924 | FSU_1373 | CBM6 | – | Yes | carbohydrate binding family | – | – |
| GH43 | Fisuc_1762 | FSU_2262 | CBM6 | BTD | No | β-xylosidase | – | – |
| GH43 | Fisuc_1763 | FSU_2263 | CBM6 | BTD | Yes | β-xylosidase | – | – |
| GH43 | Fisuc_1764 | FSU_2264 | CBM6 | BTD | Yes | β-xylosidase | – |  |
| GH43 | Fisuc_1769 | FSU_2269 | CBM6 | BTD | No | β-xylosidase | arabinoxylanase | This work |
| GH43 | Fisuc_1775 | FSU_2274 | CBM6 | BTD | Yes | β-xylosidase | – | – |
| GH43 | Fisuc_1994 | FSU_2517 | CBM35, CBM61 | – | Yes | β-xylosidase | arabinase | This work |
| GH43 | Fisuc_1997 | FSU_2520 | – | – | No | xylanase | – | – |
| GH43 | Fisuc_1998 | FSU_2521 | – | – | Yes | β-xylosidase | – | – |
| GH43 | Fisuc_1999 | FSU_2522 | CBM35 | – | No | β-xylosidase | – | – |
| GH43 | Fisuc_2621 | FSU_3190 | – | – | Yes | β-xylosidase | – | – |
| GH43 | Fisuc_2622 | FSU_3191 | CBM35 | – | Yes | β-xylosidase | – | – |
| GH43 | Fisuc_2623 | FSU_3192 | CBM35 | – | Yes | β-xylosidase | – | – |
| GH43 | Fisuc_2886 | FSU_0145 | – | – | Yes | β-xylosidase | – | – |
| GH43 | Fisuc_2929 | FSU_0192 | CBM6 | BTD | No | xylanase | – | – |
| GH44 | Fisuc_0323 | FSU_0738 | – | – | No | cellulase-like protein | – | – |
| GH45 | Fisuc_1425 | FSU_1893 | – | – | No | cellulase | cellulase |  |
| GH45 | Fisuc_1426 | FSU_1894 | – | – | No | cellulase | xylanase |  |
| GH45 | Fisuc_1473 | FSU_1947 | – | – | Yes | cellulase | – | – |
| GH45 | Fisuc_1933 | FSU_2443 | – | – | Yes | cellulase | – | – |
| GH51 | Fisuc_2081 | FSU_2610 | – | – | Yes | endoglucanase | cellulase |  |
| GH51 | Fisuc_3111 | FSU_0382 | CBM11, CBM30 | – | Yes | β-glucanase | cellulase |  |
| GH53 | Fisuc_1996 | FSU_2519 | CBM35 | – | No | arabinogalactan endo-1,4-β-galactosidase | – | – |
| GH53 | Fisuc_2461 | FSU_3024 | – | – | Yes | arabinogalactan endo-1,4-β-galactosidase | – | – |
| GH54 | Fisuc_0389 | FSU_0805 | CBM6 | – | Yes | α-N-arabinofuranosidase | – | – |
| GH57 | Fisuc_0668 | FSU_1098 | – | – | No | α-amylase | – | – |
| GH57 | Fisuc_0717 | FSU_1154 | – | – | No | α-amylase | – | – |
| GH57 | Fisuc_0731 | FSU_1169 | – | – | No | α-amylase | α-amylase |  |
| GH74 | Fisuc_2317 | FSU_2864 | – | – | Yes | xyloglucanase | – | – |
| GH77 | Fisuc_0860 | FSU_1304 | – | – | No | 4-α-glucanotransferase | – | – |
| GH94 | Fisuc_2900 | FSU_0162 | – | – | No | cellodextrin-phosphorylase | – | – |
| GH95 | Fisuc_1789 | FSU_2289 | CBM6 | – | Yes | α-L-fucosidase | none found | This work |
| GH116 | Fisuc_2704 | FSU_3272 | – | – | No | glucosylceramidase | – | – |
| NC | Fisuc_1790 | FSU_2290 | CBM6 | – | Yes | carbohydrate-binding protein | – | – |
| NC | Fisuc_2250 | FSU_2795 | – | – | Yes | xylanase | – | – |
| **Carbohydrate Esterases** | | | | | | | | |
| CE1 | Fisuc_1771 | FSU_2270 | CBM4 | BTD | Yes | esterase | esterase | This work |
| CE1 | Fisuc_1568 | FSU_2052 | – | – | Yes | esterase | – | – |
| CE1 | Fisuc_1569 | FSU_2054 | – | – | Yes | esterase | – | – |
| CE1 | Fisuc_1768 | FSU_2268 |  |  | Yes | feruloyl esterase | – | – |
| CE1 | Fisuc_1948 | FSU_2468 | – | – | Yes | esterase | – | – |
| CE1 | Fisuc_2396 |  | CBM4 | – | Yes | esterase | – | – |
| CE2 | Fisuc_1641 | FSU_2130 | – | – | Yes | esterase | esterase | This work |
| CE6 | Fisuc_1766 | FSU_2266 | CBM6 | BTD | Yes | acetyl xylan esterase | acetyl xylan esterase |  |
| CE6 | Fisuc_1767 | FSU_2267 | CBM6 | BTD | Yes | acetyl xylan esterase | acetyl xylan esterase |  |
| CE6 | Fisuc_2315 | FSU_2864 | – | – | Yes | esterase | – | – |
| CE6 | Fisuc_2534 | FSU_3103 | CBM6 | BTD | Yes | acetyl xylan esterase | xylan esterase | This work |
| CE6 | Fisuc_2800 | FSU_0054 | – | – | Yes | acetyl xylan esterase | – | – |
| CE8 | Fisuc_0679 | FSU_1115 | CBM35 | – | No | pectin esterase | – | – |
| CE12 | Fisuc_1995 | FSU_2518 | – | – | Yes | esterase | – | – |
| CE12 | Fisuc_2478 | FSU_3044 | CBM35 | – | Yes | esterase | – | – |
| CE12 | Fisuc_2479 | FSU_3045 | CBM35 | – | No | esterase | – | – |
| CE15 | Fisuc_2348 | FSU_2898 | – | – | No | esterase | – | – |
| **Polysaccharide Lyases** | | | | | | | | |
| PL1 | Fisuc_0174 | FSU_0577 | – | – | Yes | pectate lyase | – | – |
| PL1 | Fisuc_1991 | FSU_2514 | – | BTD | Yes | pectate lyase | – | – |
| PL1 | Fisuc_2002 | FSU_2525 | – | – | No | pectate lyase | – | – |
| PL1 | Fisuc_2012 | FSU_2535 | – | – | No | pectate lyase | – | – |
| PL1 | Fisuc_2363 | FSU_2913 | – | – | Yes | pectate lyase | – | – |
| PL1 | Fisuc_2481 | FSU_3048 | – | – | Yes | pectate lyase | – | – |
| PL1 | Fisuc_2565 | FSU_3135 | CBM35 | BTD | Yes | pectate lyase | – | – |
| PL9 | Fisuc_1993 | FSU_2516 | – | – | No | pectate lyase | – | – |
| PL10 | Fisuc_0678 | FSU_1114 | CBM35 | – | Yes | pectate lyase | cellulase | This work |
| PL11 | Fisuc_2005 | FSU_2528 | – | – | Yes | pectate lyase | – | – |
| PL11 | Fisuc_2477 | FSU_3043 | CBM35 | – | Yes | pectate lyase | – | – |
| PL14 | Fisuc_0498 | FSU_0916 | – | – | Yes | pectate lyase | – | – |
| **Carbohydrate Binding Modules**^d^ | | | | | | | | |
| CBM4 | Fisuc_1931 | FSU_2441 | CBM4 | – | No | carbohydrate binding module | – | – |
| CBM6 | Fisuc_1774 | FSU_2273 | CBM6 | – | Yes | carbohydrate binding module | – | – |
| CBM6 | Fisuc_2485 | FSU_3051 | CBM6 | BTD | Yes | carbohydrate binding module | none found | This work |
| CBM30 | Fisuc_1525 | FSU_2007 | CBM30 | – | Yes | carbohydrate binding module | – | – |
| CBM51 | Fisuc_0215 | FSU_0622 | CBM51 | – | No | carbohydrate binding module | – | – |
| CBM51 | Fisuc_0401 | FSU_0816 | CBM51 | – | No | carbohydrate binding module | – | – |
| CBM51 | Fisuc_1656 | FSU_2145 | CBM51 | – | Yes | carbohydrate binding module | – | – |

^a^ NC, non classified CAZyme families

^b^ FSU locus tags refer to the equivalent ORF call in the *F. succinogenes* genome sequence project described by GenBank accession: CP002158

^c^ Basic Terminal Domain (BTD) is also known as *F. succinogenes*-specific paralogous module 1 (FPm-1) .

^d^ Only genes predicted to encode only CBMs are indicated. CBMs existing on other CAZymes are listed under CBM Family.

**References**

1. Iyo AH, Forsberg CW (1996) Endoglucanase G from *Fibrobacter succinogenes* S85 belongs to a class of enzymes characterized by a basic C-terminal domain. Can J Microbiol 42: 934-943.

2. Iyo AH, Forsberg CW (1994) Features of the cellodextrinase gene from *Fibrobacter succinogenes* S85. Can J Microbiol 40: 592-596.

3. McGavin MJ, Forsberg CW, Crosby B, Bell AW, Dignard D, et al. (1989) Structure of the cel-3 gene from *Fibrobacter succinogenes* S85 and characteristics of the encoded gene product, endoglucanase 3. J Bacteriol 171: 5587-5595.

4. Qi M, Jun H-S, Forsberg CW (2007) Characterization and synergistic interactions of *Fibrobacter succinogenes* glycoside hydrolases. Appl Environ Microbiol 73: 6098-6105.

5. Brumm P, Mead D, Boyum J, Drinkwater C, Gowda K, et al. (2010) Functional annotation of *Fibrobacter succinogenes* S85 carbohydrate active enzymes. Appl Biochem Biotechnol: 10.1007/s12010-12010-19070-12015.

6. Broussolle V, Forano E, Gaudet G, Ribot Y (1994) Gene sequence and analysis of protein domains of EGB, a novel family E endoglucanase from *Fibrobacter succinogenes* S85. FEMS Microbiol Lett 124: 439-447.

7. Malburg LM, Jr., Iyo AH, Forsberg CW (1996) A novel family 9 endoglucanase gene (celD), whose product cleaves substrates mainly to glucose, and its adjacent upstream homolog (celE) from *Fibrobacter succinogenes* S85. Appl Environ Microbiol 62: 898-606.

8. Qi M, Jun H-S, Forsberg CW (2008) Cel9D, an atypical 1,4-β-D-glucan glucohydrolase from *Fibrobacter succinogenes*: characteristics, catalytic residues, and synergistic interactions with other cellulases. J Bacteriol 190: 1976-1984.

9. Cavicchioli R, Watson K (1991) Molecular cloning, expression, and characterization of endoglucanase genes from *Fibrobacter succinogenes* AR1. Appl Environ Microbiol 57: 359-365.

10. Jun H-S, Ha JK, Malburg LM, Jr., Verrinder GA, Forsberg CW (2003) Characteristics of a cluster of xylanase genes in *Fibrobacter succinogenes* S85. . Can J Microbiol 49: 171-180.

11. Jun H-S, Qi M, Gong J, Egbosimba EE, Forsberg CW (2007) Outer membrane proteins of *Fibrobacter succinogenes* with potential roles in adhesion to cellulose and in cellulose digestion. J Bacteriol 189: 6806-6815.

12. Paradis FW, Zhu H, Krell PJ, Phillips JP, Forsberg CW (1993) The xynC gene from *Fibrobacter succinogenes* S85 codes for a xylanase with two similar catalytic domains. J Bacteriol 175: 7666-7672.

13. Teather RM, Erfle JD (1990) DNA sequence of a *Fibrobacter succinogenes* mixed-linkage beta-glucanase (1,3-1,4-beta-D-glucan 4-glucanohydrolase) gene. J Bacteriol 172: 3837-3841.

14. Mitsumori M, Minato H, Sekizaki T, Uchida I, Ito H (1996) Cloning, nucleotide sequence and expression of the gene encoding the cellulose-binding protein 1 (CBP1) of *Fibrobacter succinogenes* S85. FEMS Microbiol Lett 139: 43-50.

15. Malburg SR, Malburg LM, Jr., Liu T, Iyo AH, Forsberg CW (1997) Catalytic properties of the cellulose-binding endoglucanase F from *Fibrobacter succinogenes* S85. Appl Environ Microbiol 63: 2449-2453.

16. Kam DK, Jun H-S, Ha JK, Inglis GD, Forsberg CW (2005) Characteristics of adjacent family 6 acetylxylan esterases from *Fibrobacter succinogenes* and the interaction with the Xyn10E xylanase in hydrolysis of acetylated xylan. Can J Microbiol 51: 821-883.

17. Yoshida S, Mackie RI, Cann IKO (2010) Biochemical and domain analyses of FSUAxe6B, a modular acetyl xylan esterase, identify a unique carbohydrate binding module in *Fibrobacter succinogenes* S85. J Bacteriol 192: 483-493.
