# Supplementary material for: The Complete Genome Sequence of Fibrobacter succinogenes S85 Reveals a Cellulolytic and Metabolic Specialist
Source: PLoS One. 2011 Apr 19;6(4):e18814. doi: 10.1371/journal.pone.0018814 (PMC3079729; doi:10.1371/journal.pone.0018814)
Supplement: Table S3 — Comparison of carbohydrate-degrading enzymes (CAZymes) encoded by the genomes of the 4 ruminant bacteria Fibrobacter succinogenes S85 (Fsuc), Butryvibrio proteoclasticus B316 (Bpro), Prevotella ruminocola 23 (Prum), and Ruminococcus flavefaciens FD-1 (Rfla). (DOC) [file pone.0018814.s003.doc]

**Table S3.** Comparison of carbohydrate-degrading enzymes (CAZymes) encoded by the genomes of the 4 ruminant bacteria *Fibrobacter succinogenes* S85 (Fsuc), *Butryvibrio proteoclasticus* B316 (Bpro), *Prevotella ruminocola* 23 (Prum), and *Ruminococcus flavefaciens* FD-1 (Rfla).

| **CAZy Family** | **Known Activities** | **Fsuc** | **Bpro^c^** | **Prum^a^** | **Rfla^b^** |
| --- | --- | --- | --- | --- | --- |
| CBM2 | Binding has been demonstrated with cellulose, chitin, and xylan | 0 | 9 | 0 | 0 |
| CBM3 | Binding has been demonstrated with cellulose and chitin | 0 | 1 | 0 | 5 |
| CBM4 | Binding has been demonstrated with xylan, β-1,3-glucan, β-1,3-1,4-glucan, β-1,6-glucan and amorphous cellulose | 4 | 0 | 0 | 7 |
| CBM6 | Binding on amorphous cellulose and β-1,4-xylan, β-1,3-glucan, β-1,3-1,4-glucan, and β-1,4-glucan | 20 | 5 | 3 | 3 |
| CBM9 | Found to be associated with xylanases | 0 | 2 | 0 | 0 |
| CBM11 | Binds both β-1,4-glucan and β-1,3-1,4-mixed linked glucans. | 1 | 0 | 0 | 0 |
| CBM13 | Binding to xylan demonstrated in *Streptomyces* | 0 | 3 | 1 | 10 |
| CBM20 | Starch binding domain | 0 | 0 | 4 | 0 |
| CBM26 | Starch-binding domain | 0 | 2 | 0 | 0 |
| CBM30 | Binding to cellulose in *Fibrobacter succinogenes* CelF. | 3 | 0 | 0 | 0 |
| CBM32 | Binding has been demonstrated with galactose and lactose | 0 | 0 | 4 | 3 |
| CBM34 | Granular starch-binding demonstrated for *Thermoactinomyces vulgaris* R-47 | 0 | 2 | 0 | 0 |
| CBM35 | A module that is conserved in 3 *Cellvibrio* xylan-degrading enzymes | 14 | 0 | 1 | 13 |
| CBM36 | Calcium-dependent binding to xylan demonstrated for *Paenbacillus polymyxa* | 0 | 1 | 0 | 0 |
| CBM41 | Binding has been demonstrated in  *Thermotoga maritima* Pul13 for α-glucans amylose, amylopectin, pullulan, and oligosaccharide fragments derived from these polysaccharides | 0 | 1 | 0 | 0 |
| CBM48 | Modules appended to GH13 modules | 1 | 4 | 1 | 2 |
| CBM50 | Modules attached to GH18, GH19, GH23, GH24, GH25 and GH73 | 6 | 0 | 2 | 0 |
| CBM51 | Modules attached to GH2, GH27, GH31, GH95, GH98 and GH101 | 3 | 0 | 0 | 0 |
| CBM61 | Modules attached to GH16, GH30, GH31, GH43, GH53 and GH66 catalytic domains | 1 | 1 | 0 | 0 |
| GH1 | β-glucosidase; β-galactosidase; β-mannosidase | 0 | 1 | 0 | 0 |
| GH2 | β-galactosidase ; β-mannosidase; β-glucuronidase | 2 | 9 | 10 | 2 |
| GH3 | β-glucosidase; xylan 1,4-β-xylosidase; β-N-acetylhexosaminidase | 3 | 10 | 11 | 6 |
| GH5 | chitosanase; β-mannosidase; Cellulase | 12 | 4 | 3 | 14 |
| GH8 | chitosanase; cellulase; endo-1,4-β-xylanase | 6 | 1 | 1 | 0 |
| GH9 | endoglucanase; cellobiohydrolase; β-glucosidase | 9 | 3 | 0 | 12 |
| GH10 | endo-1,4-β-xylanase; endo-1,3-β-xylanase | 7 | 6 | 2 | 6 |
| GH11 | xylanase | 4 | 0 | 0 | 11 |
| GH13 | α-amylase; pullulanase; cyclomaltodextrin glucanotransferase | 3 | 14 | 5 | 4 |
| GH16 | xyloglucan:xyloglucosyltransferase; keratan-sulfate endo-1,4-β-galactosidase | 4 | 2 | 2 | 5 |
| GH18 | chitinase; endo-β-N-acetylglucosaminidase | 2 | 1 | 1 | 1 |
| GH20 | β-hexosaminidase; lacto-N-biosidase; β-1,6-N-acetylglucosaminidase); β-6-SO3-N-acetylglucosaminidase | 0 | 0 | 2 | 0 |
| GH23 | lysozyme type G | 3 | 0 | 2 | 0 |
| GH24 | lysozyme | 0 | 0 | 1 | 1 |
| GH25 | lysozyme | 0 | 5 | 3 | 9 |
| GH26 | β-mannanase; β-1,3-xylanase | 5 | 0 | 1 | 6 |
| GH27 | α-galactosidase; α-N-acetylgalactosaminidase; isomalto-dextranase | 1 | 2 | 1 | 0 |
| GH28 | polygalacturonase; exo-polygalacturonase; exo-polygalacturonosidase | 0 | 2 | 5 | 0 |
| GH29 | α-L-fucosidase | 0 | 2 | 3 | 0 |
| GH30 | glucosylceramidase; β-1,6-glucanase; β-xylosidase | 3 | 2 | 0 | 0 |
| GH31 | α-glucosidase; α-1,3-glucosidase; sucrase-isomaltase | 0 | 5 | 6 | 1 |
| GH32 | invertase; endo-inulinase; β-2,6-fructan 6-levanbiohydrolase; endo-levanase | 0 | 3 | 2 | 0 |
| GH35 | β-galactosidase; exo-β-glucosaminidase | 0 | 2 | 2 | 0 |
| GH36 | α-galactosidase; α-N-acetylgalactosaminidase; stachyose synthase | 0 | 3 | 1 | 1 |
| GH38 | α-mannosidase; mannosyl-oligosaccharide α-1,3-1,6-mannosidase; mannosyl-oligosaccharide α-1,3-mannosidase | 0 | 1 | 1 | 0 |
| GH39 | α-L-iduronidase; β-xylosidase | 1 | 1 | 0 | 0 |
| GH43 | β-xylosidase; β-1,3-xylosidase; α-L-arabinofuranosidase | 14 | 11 | 20 | 10 |
| GH44 | endoglucanase; xyloglucanase | 1 | 0 | 0 | 2 |
| GH45 | Endoglucanase | 4 | 0 | 0 | 0 |
| GH48 | endoglucanase; chitinase; cellobiohydrolase; endo-processive cellulases | 0 | 0 | 0 | 1 |
| GH51 | α-L-arabinofuranosidase; endoglucanase | 2 | 2 | 7 | 0 |
| GH53 | endo-β-1,4-galactanase | 2 | 2 | 2 | 1 |
| GH54 | α-L-arabinofuranosidase; β-xylosidase | 1 | 0 | 0 | 0 |
| GH55 | exo-β-1,3-glucanase; endo-β-1,3-glucanase | 0 | 1 | 0 | 0 |
| GH57 | α-amylase; 4-α-glucanotransferase; α-galactosidase | 3 | 0 | 1 | 0 |
| GH67 | α-glucuronidase; xylan α-1,2-glucuronosidase | 0 | 1 | 1 | 0 |
| GH73 | peptidoglycan hydrolase with endo-β-N-acetylglucosaminidase specificity | 0 | 0 | 1 | 0 |
| GH74 | endoglucanase; xyloglucanase | 1 | 0 | 0 | 1 |
| GH76 | α-1,6-mannanase | 0 | 0 | 1 | 0 |
| GH77 | amylomaltase or 4-α-glucanotransferase | 1 | 1 | 2 | 1 |
| GH78 | α-L-rhamnosidase | 0 | 4 | 1 | 0 |
| GH88 | d-4,5 unsaturated β-glucuronyl hydrolase | 0 | 1 | 0 | 0 |
| GH89 | α-N-acetylglucosaminidase | 0 | 0 | 1 | 0 |
| GH92 | mannosyl-oligosaccharide α-1,2-mannosidase; mannosyl-oligosaccharide α-1,3-mannosidase | 0 | 0 | 8 | 0 |
| GH94 | cellobiose phosphorylase; cellodextrin phosphorylase; chitobiose phosphorylase | 1 | 2 | 1 | 1 |
| GH95 | α-1,2-L-fucosidase; α-L-fucosidase | 1 | 1 | 2 | 1 |
| GH97 | α-glucosidase; α-galactosidase | 0 | 0 | 6 | 2 |
| GH105 | unsaturated rhamnogalacturonyl hydrolase | 0 | 3 | 2 | 1 |
| GH106 | α-L-rhamnosidase | 0 | 0 | 1 | 0 |
| GH115 | xylan α-1,2-glucuronosidase; α-glucuronidase | 0 | 1 | 1 | 0 |
| GH116 | β-glucosidase; β-xylosidase | 1 | 3 | 0 | 0 |
| CE1 | acetyl xylan esterase; cinnamoyl esterase; feruloyl esterase | 6 | 4 | 4 | 8 |
| CE2 | acetyl xylan esterase | 1 | 1 | 1 | 3 |
| CE3 | acetyl xylan esterase | 0 | 0 | 0 | 3 |
| CE4 | acetyl xylan esterase; chitin deacetylase; chitooligosaccharide deacetylase | 0 | 1 | 1 | 5 |
| CE6 | acetyl xylan esterase | 5 | 0 | 1 | 0 |
| CE7 | acetyl xylan esterase; cephalosporin-C deacetylase | 0 | 0 | 2 | 0 |
| CE8 | pectin methylesterase | 1 | 2 | 2 | 1 |
| CE9 | N-acetylglucosamine 6-phosphate deacetylase; N-acetylgalactosamine-6-phosphate deacetylase | 0 | 1 | 0 | 0 |
| CE10 | arylesterase; carboxyl esterase; acetylcholinesterase | 0 | 1 | 0 | 0 |
| CE11 | UDP-3-0-acyl N-acetylglucosamine deacetylase | 0 | 0 | 1 | 1 |
| CE12 | pectin acetylesterase; rhamnogalacturonan acetylesterase; acetyl xylan esterase | 3 | 0 | 4 | 5 |
| CE15 | 4-O-methyl-glucuronoyl methylesterase | 1 | 0 | 1 | 1 |
| PL1 | pectate lyase; exo-pectate lyase; pectin lyase | 7 | 1 | 1 | 6 |
| PL9 | pectate lyase; exopolygalacturonate lyase | 1 | 2 | 0 | 1 |
| PL10 | pectate lyase | 1 | 0 | 1 | 0 |
| PL11 | rhamnogalacturonan lyase | 2 | 1 | 1 | 6 |
| PL14 | alginate lyase | 1 | 0 | 0 | 0 |

^a^ Obtained from CAZy (http://CAZy.org)

^b^ Obtained from: Berg Miller ME, Antonopoulos DA, Rincon MT, Band M, Bari A, et al. (2009) Diversity and strain specificity of plant cell wall degrading enzymes revealed by the draft genome of *Ruminococcus flavefaciens* FD-1. PLoS ONE 4: e6650.

^c^ Obtained from CAZy (http://CAZy.org)
