# Supplementary material for: The Complete Genome Sequence of Fibrobacter succinogenes S85 Reveals a Cellulolytic and Metabolic Specialist
Source: PLoS One. 2011 Apr 19;6(4):e18814. doi: 10.1371/journal.pone.0018814 (PMC3079729; doi:10.1371/journal.pone.0018814)
Supplement: Table S4 — Primers used to clone selected CAZymes in F. succinogenes and their tested polysaccharide activity. (DOC) [file pone.0018814.s004.doc]

**Table S4.** Primers used to clone selected CAZymes in *F. succinogenes* and their tested polysaccharide activity.

| **Gene** | **Forward Primer (5’ to 3’)** | **Reverse Primer (5’ to 3’)** | ***endo*-glucanase activity^1^** | ***exo*-glucanase activity^1^** |
| --- | --- | --- | --- | --- |
| Fisuc_0241 | GAAGGAGATATACATATGTTGCAACGCTACCCGACGCGAGAT | GTGATGGTGGTGATGATGGAGTGTGATTTTTCCCGCGGTGGCGAGT | **HEC** | **MUC** |
| Fisuc_0727 | GAAGGAGATATACATATGCCATATGAAGCCGAAGACGCAACCATC | GAAGGAGATATACATATGCCATATGAAGCCGAAGACGCAACCATC | **GM** | **None** |
| Fisuc_0728 | GTGATGGTGGTGATGATGCTTCACAATTACGGGCTGCGTCGTGGTGAT | GTGATGGTGGTGATGATGCTTCACAATTACGGGCTGCGTCGTGGTGAT | **GM** | **MUC** |
| Fisuc_0786 | GAAGGAGATATACATATGGCCCTCCCCAAGGCAACCGCATTGGTCGA | GTGATGGTGGTGATGATGGCGGAGGCGGACTTGCTGTACGCTCTTCTG | **HEC** | **None** |
| Fisuc_1224 | GAAGGAGATATACATATGGATGCATCGACAGCAACCCCCAAGAAAGT | GAAGGAGATATACATATGGATGCATCGACAGCAACCCCCAAGAAAGT | **BG** | **None** |
| Fisuc_1523 | GAAGGAGATATACATATGGCAACAATACCGACATCGGCCGGCAAGGGCA | GTGATGGTGGTGATGATGGAAGATGAGGGCTTTAGGGAATCCTTCGT | **HEC** | **MUC** |
| Fisuc_1641 | GAAGGAGATATACATATGGAAAACCTTCTGTTCAATGGCCGCT | GAAGGAGATATACATATGGAAAACCTTCTGTTCAATGGCCGCT | **None** | **PNPA** |
| Fisuc_1769 | GAAGGAGATATACATATGGTTAAGGTCAATAACCCGATCATG | GTGATGGTGGTGATGATGCTTTGTTACGGATAGGCGGTGGGTG | **AX** | **MUX** |
| Fisuc_1771 | GAAGGAGATATACATATGGATCAGGCTACATTCTATGTCGCTCCC | GTGATGGTGGTGATGATGCTTCACCTGAATCATTTTGCCAAATGCCT | **None** | **PNPA** |
| Fisuc_1789 | GAAGGAGATATACATATGACGGACAATCCGCTTACGCTTTGG | GTGATGGTGGTGATGATGATGGACAGTGAGTTTCATGGTCTT | **None** | **None** |
| Fisuc_1994 | GAAGGAGATATACATATGTTTGGATTGAGAGAAATCTTCAAA | GTGATGGTGGTGATGATGTTTTGAATACACTCTAATTCTGGAAGCT | **AR** | **MUC** |
| Fisuc_2065 | GAAGGAGATATACATATGGGAATTAGCGGTTCTATCGTTGATGAATCC | GTGATGGTGGTGATGATGGTATGTCAACCCGAATCCGTACGGGTAGAG | **HEC** | **MUC** |
| Fisuc_2485 | GAAGGAGATATACATATGCCGATCACTACGGTTCCTTGGAATGGT | GAAGGAGATATACATATGCCGATCACTACGGTTCCTTGGAATGGT | **None expected (CBM)** | **None expected (CBM)** |
| Fisuc_2534 | GAAGGAGATATACATATGCCGGACCCGAATTTCCACATCTACATT | GTGATGGTGGTGATGATGCTTCATCACACGGATGGTCGCGGAGCT | **AX** | **PNPA** |
| Fisuc_2933 | GAAGGAGATATACATATGGAGAAAATTAAAGACATGAACGGCT | GTGATGGTGGTGATGATGTCCGAGGTCTTTGATGACCATGATACC | **GM** | **None** |
| Fisuc_3049 | GTGATGGTGGTGATGATGCAGCAAGTTCACGATTGCCGTGAAC | GTGATGGTGGTGATGATGCAGCAAGTTCACGATTGCCGTGAAC | **None** | **XG** |
| Fisuc_3081 | GAAGGAGATATACATATGGATTTGCCGACGGCAAACGAAATGTTT | GAAGGAGATATACATATGGATTTGCCGACGGCAAACGAAATGTTT | **HEC AX** | **none** |

**^1^ AR**- AZCL-Arabinan, **AX**-AZCL-arabinoxylan, **BG**–AZCL-*beta*-glucan, , **GM**- AZCL-galactomannan, **HEC**-AZCL-HE cellulose**, MUC-** methylumbelliferyl-β-D-cellobioside,  **MUX**-4-methylumbelliferyl-β-D-xylopyranoside, **, XG**-5-Bromo-4-chloro-3-indolyl β-D-galactopyranoside, **XYG**- AZCL-Xyloglucan, **PNPA**- p-nitrophenyl acetate.
