# Supplementary material for: The Complete Genome Sequence of Fibrobacter succinogenes S85 Reveals a Cellulolytic and Metabolic Specialist
Source: PLoS One. 2011 Apr 19;6(4):e18814. doi: 10.1371/journal.pone.0018814 (PMC3079729; doi:10.1371/journal.pone.0018814)
Supplement: Text S1 — Glycogen biosynthesis and utilization. (DOC) [file pone.0018814.s005.doc]

**Text S1: Glycogen biosynthesis and utilization.**

Production of glycogen by *F. succinogenes* as a reservoir for energy and carbon is well known , and physiological studies have revealed that glycogen appears to be simultaneously synthesized and degraded in a futile cycle. The physiological purpose of this cycling is unknown. The carbohydrate-active enzyme (CAZy) database annotates 54 glycosyl transferases (GTs) (Table 1) in the genome of *F. succinogenes.* A number of these GTs appear in gene clusters (Fisuc_0502 - Fisuc_0504, Fisuc_0709 - Fisuc_0713, Fisuc_2652 - Fisuc_2659, and Fisuc_2740-2742). Without clear biochemical or genetic characterization of the individual GT family members, it is premature to assign functions in glycogen biosynthesis to any individual GT. Hydrolysis of glycogen to glucose is most likely accomplished using a combination of glycosyl hydrolase 13 (Fisuc_0859, Fisuc_1932, and Fisuc_3103) and 57 (Fisuc_0688, Fisuc_0717, and Fisuc_0731) family members. These proteins all share a lack of a signal peptide, indicating an intracellular localization and, combined with the inability of *F. succinogenes* to degrade exogenously supplied starch , suggests that glycogen metabolism is the most likely role for these proteins.

**References**

1. Gaudet G, Forano E, Dauphin G, Delort AM (1992) Futile cycling of glycogen in *Fibrobacter succinogenes* as shown by in situ 1H-NMR and 13C-NMR investigation. Eur J Biochem 207: 155-162.

2. Gong J, Forsberg CW (1993) Separation of outer and cytoplasmic membranes of *Fibrobacter succinogenes* and membrane and glycogen granule locations of glycanases and cellobiase. J Bacteriol 175: 6810-6821.

3. Matheron C, Delort A-M, Gaudet G, Forano E, Liptaj T (1998) 13C and 1H nuclear magnetic resonance study of glycogen futile cycling in strains of the genus *Fibrobacter*. Appl Environ Microbiol 64: 74-81.

4. Cantarel BL, Coutinho PM, Rancurel C, Bernard T, Lombard V, et al. (2009) The Carbohydrate-Active EnZymes database (CAZy): an expert resource for glycogenomics. Nucleic Acids Res 37: D233-238.

5. Hungate RE (1950) The anaerobic mesophilic cellulolytic bacteria. Bacteriol Rev 14: 1-49.

**Table 1**. Glycosyl transferase (GT) genes in *Fibrobacter succinogenes* S85 with loci in bold representing putative operons.

| **Gene** | **GT Family** |
| --- | --- |
| Fisuc_0062 | GT51 |
| Fisuc_0083 | GT2 |
| Fisuc_0097 | GT2 |
| Fisuc_0214 | NC |
| Fisuc_0402 | GT2 |
| Fisuc_0443 | GT4 |
| **Fisuc_0502** | GT2 |
| **Fisuc_0503** | GT2 |
| **Fisuc_0504** | GT2 |
| Fisuc_0534 | GT2 |
| Fisuc_0566 | GT28 |
| Fisuc_0687 | GT2 |
| Fisuc_0694 | GT4 |
| **Fisuc_0709** | GT4 |
| **Fisuc_0711** | GT32 |
| **Fisuc_0712** | GT2 |
| Fisuc_0823 | GT1 |
| Fisuc_0865 | GT19 |
| Fisuc_0953 | GT26 |
| **Fisuc_0970** | GT2 |
| **Fisuc_0971** | GT2 |
| **Fisuc_0983** | GT32 |
| **Fisuc_0984** | GT2 |
| Fisuc_1252 | GT51 |
| Fisuc_1439 | GT4 |
| Fisuc_1454 | NC |
| Fisuc_1515 | GT4 |
| Fisuc_1521 | GT2 |
| Fisuc_1578 | GT2 |
| Fisuc_1661 | GT2 |
| Fisuc_1939 | GT30 |
| Fisuc_1987 | GT2 |
| **Fisuc_2045** | GT32 |
| **Fisuc_2046** | GT2 |
| **Fisuc_2048** | GT2 |
| Fisuc_2058 | GT2 |
| Fisuc_2067 | GT5 |
| Fisuc_2097 | GT35 |
| Fisuc_2240 | GT4 |
| Fisuc_2310 | GT2 |
| **Fisuc_2652** | GT4 |
| **Fisuc_2653** | GT4 |
| **Fisuc_2655** | GT4 |
| **Fisuc_2656** | GT2 |
| **Fisuc_2659** | GT2 |
| **Fisuc_2674** | GT2 |
| **Fisuc_2676** | GT4 |
| **Fisuc_2679** | GT2 |
| **Fisuc_2680** | GT4 |
| **Fisuc_2684** | GT4 |
| **Fisuc_2740** | GT2 |
| **Fisuc_2741** | GT2 |
| **Fisuc_2742** | GT2 |
| Fisuc_3008 | GT2 |
