# Supplementary material for: The Complete Genome Sequence of Fibrobacter succinogenes S85 Reveals a Cellulolytic and Metabolic Specialist
Source: PLoS One. 2011 Apr 19;6(4):e18814. doi: 10.1371/journal.pone.0018814 (PMC3079729; doi:10.1371/journal.pone.0018814)
Supplement: Text S2 — Amino acid synthesis and nitrogen assimilation. (DOC) [file pone.0018814.s006.doc]

**Text S2: Amino acid synthesis and nitrogen assimilation**

Based on our metabolic reconstruction of the genome, *F. succinogenes* possesses complete pathways for the de novo synthesis of both aromatic (histidine, phenylalanine, tyrosine, and tryptophan) and aliphatic (cysteine, methionine, leucine and isoleucine) amino acids from pyruvate and ammonia. *F. succinogenes* appears to not synthesize isoleucine through the conversion of threonine, but uses two other pathways. The first is through the conversion of propionate into 2-ketobutanoate, which serves as the starting substrate for isoleucine biosynthesis. A second recently-reported pathway is based on the synthesis of citramalate from acetyl-CoA and pyruvate that is catalyzed by a citramalate synthase (*cimA*, Fisuc_2757). Citramalate is then converted into D-erythro-3-methylmalate through the action of LeuCD (Fisuc_0067-Fisuc_0068). This, in turn, is converted into 2-ketobutanoate through a 3-isopropylmalate dehydrogenase (*leuB*, Fisuc_2644).

This pattern of amino acid synthesis in *F. succinogenes* is in agreement with the known requirement for ammonia as a nitrogen source and the lack of a requirement for preformed amino acids in chemically defined medium . *F. succinogenes* appears to have a limited ability to use preformed amino acids when supplemented in the medium , and it has been reported that it consumes several-fold more nitrogen than can be accounted for by cellular nitrogen, with the difference being released by cell lysis . The precise physiological and environmental significance of this is unknown. Based on the metabolic reconstruction, the organism appears, like other ruminal cellulolytic bacteria, to lack the ability to catabolize these amino acids to produce ammonia and glycolytic or Krebs cycle intermediates. The inability of the organism to generate these intermediates may partially contribute to its rapid death when depleted of ammonia .

Ammonia appears to be the primary mechanism through which *F. succinogenes* acquires nitrogen, as it does not have the genes necessary for the utilization or uptake of either nitrate or nitrite. Ammonia assimilation occurs through the action of at least 4 enzymes, including a glutamate synthetase (Fisuc_1120 and Fisuc_1816), a glutamate dehydrogenase (Fisuc_2811), a histidine ammonia lyase (Fisuc_3006), and a carbamoyl-phosphate synthase (Fisuc_0813, Fisuc_0835, and Fisuc_2938). Ammonium uptake in *F. succinogenes* appears to be coupled to a single high-affinity ammonium transporter (*amtB*, Fisuc_1820) that is found as part of the *glnKamtB* operon . The transcriptional regulator GlnK is known to regulate the level of glutamine synthetase in bacterial cells in response to nitrogen-limiting conditions, and a glutamine synthetase operon is found upstream of this ammonium transporter.

*F. succinogenes* also contains a number of nitrogenase genes, suggesting the ability to fix nitrogen; however, nitrogen-fixing capability has never been demonstrated in this organism. Analysis of the genetic region containing these genes reveals a cluster of 29 genes (Fisuc_1074 – Fisuc1102), but does not contain the full complement of proteins in the canonical *nif* pathway common to other bacteria like *Klebsiella* and *Enterobacter*. This cluster does bear similarity to a nitrogenase cluster in *Clostridium beijerinckii* NCIMB 8052 . The function of this *C. beijerinckii* nitrogenase cluster is unknown, as it contains a second cluster of nitrogenase genes known to be responsible for nitrogen fixation in this species. These data indicate that perhaps *F. succinogenes* had a prior ability to fix nitrogen, but may have lost this capacity due to the abundance of ammonia present in the rumen.

**References**

1. Howell DM, Xu H, White RH (1999) (R)-Citramalate synthase in methanogenic archaea. J Bacteriol 181: 331-333.

2. Wells JE, Russell JB (1996) The effect of growth and starvation on the lysis of the ruminal cellulolytic bacterium *Fibrobacter succinogenes*. Appl Environ Microbiol 62: 1342-1346.

3. Atasoglu C, Newbold CJ, Wallace RJ (2001) Incorporation of [15N]ammonia by the cellulolytic ruminal bacteria *Fibrobacter succinogenes* BL2, *Ruminococcus albus* SY3, and *Ruminococcus flavefaciens* 17. Appl Environ Microbiol 67: 2819-2822.

4. Thomas S, Russell JB (2004) The effect of cellobiose, glucose, and cellulose on the survival of *Fibrobacter succinogenes* A3C cultures grown under ammonia limitation. Curr Microbiol 48: 219-223.

5. Thomas G, Coutts G, Merrick M (2000) The glnKamtB operon: a conserved gene pair in prokaryotes. Trends Genet 16: 11-14.

6. Chen JS, Toth J, Kasap M (2001) Nitrogen-fixation genes and nitrogenase activity in *Clostridium acetobutylicum* and *Clostridium beijerinckii*. J Ind Microbiol Biotechnol 27: 281-286.
