# Supplementary material for: The Complete Genome Sequence of Fibrobacter succinogenes S85 Reveals a Cellulolytic and Metabolic Specialist
Source: PLoS One. 2011 Apr 19;6(4):e18814. doi: 10.1371/journal.pone.0018814 (PMC3079729; doi:10.1371/journal.pone.0018814)
Supplement: Text S3 — Fatty acid synthesis and catabolism. (DOC) [file pone.0018814.s007.doc]

**Text S3: Fatty acid synthesis and catabolism**

*Fibrobacter succinogenes* S85 is able to synthesize fatty acids de novo from acetyl-CoA and incorporate them into phospholipids. This strain has an absolute requirement for several volatile acids for growth , utilizing isobutyrate and valerate for production of phospholipid molecules containing branched and odd carbon length fatty acids. The absence of enzymes in the butanoate and propanoate biosynthetic pathways explains the need for fatty acid precursors. No obvious genes for transport or incorporation of these two fatty acid precursors are apparent from the metabolic reconstruction, suggesting that they may enter the cell in their protonated (uncharged) forms by passive diffusion. As in amino acid metabolism, *F. succinogenes* appears to be unable to degrade fatty acids to generate metabolic intermediates.

Biosynthesis of isoprenoid derivatives is accomplished using a complete methyl erythritol phosphate (MEP) pathway; none of the enzymes of the mevalonate (MEV) pathway are present in the organism. Pathway enzymes appear present for the conversion of isopentenyl pyrophosphate dimethylallyl pyrophosphate into both squalene and hexaprenyl pyrophosphate, but not into sterols.

**References**

1. Bryant MP, Doetsch RN (1954) Factors necessary for the growth of *Bacteroides succinogenes* in the volatile acid fraction of rumen fluid. Science 120: 944-945.

2. Wegner GH, Foster EM (1963) Incorporation of isobutyrate and valerate into cellular plasmalogen by *Bacteroides succinogenes*. J Bacteriol 85: 53-61.
