# Supplementary material for: The Complete Genome Sequence of Fibrobacter succinogenes S85 Reveals a Cellulolytic and Metabolic Specialist
Source: PLoS One. 2011 Apr 19;6(4):e18814. doi: 10.1371/journal.pone.0018814 (PMC3079729; doi:10.1371/journal.pone.0018814)
Supplement: Text S4 — Vitamin biosynthesis. (DOC) [file pone.0018814.s008.doc]

**Text S4: Vitamin biosynthesis**

A cluster of genes responsible for heme, porphyrin, and cobalamin biosynthesis was reported to be present in *F. succinogenes* but is either missing from or not functional in *F. intestinalis* DR7 . The metabolic reconstruction of *F. succinogenes* shows the key intermediate in the pathway, δ-aminolevulinic acid (ALA), is produced from glutamic semialdehyde via a Glu-tRNA and not via the condensation of succinyl-CoA and glycine, as would be expected for a succinate-producing organism. The reconstruction identifies enzymes responsible for production of intermediates through precorrin 2, but does not identify the enzymes responsible for the remaining steps in corrinoid biosynthesis. Complete pathways for the production of biotin, pantothenate, folate and nicotinate are present in the reconstruction.

Further analysis of the electron transport system in the *F. succinogenes* genome reveals that it does not have the complete repertoire of genes necessary for the production of either ubiquinone or menaquinone through the *ubi* or *men* pathways, respectively. Recently, it was reported that menaquinone production can occur through an alternative pathway in organisms that do not possess the *men* pathway . A search of the four genes implicated in this alternative pathway in the *F. succinogenes* genome revealed corresponding putative homologs, suggesting that *F. succinogenes* may generate menaquinone using this alternative pathway. Under this model, chorismate is first converted to futalosine using the enzyme encoded by Fisuc_1586, then converted to de-hypoxanthine futalosine through the action of a second enzyme (Fisuc_1888), then cyclic de-hypoxanthine futalosine (Fisuc_2321), before conversion to 1,4-dihydroxy-6-naphthoate (Fisuc_1889). This last compound is then converted into menaquinone following conversion by MenA (Fisuc_0050) and MenG (Fisuc_1908), the only two genes found in *F. succinogenes* that are part of the *men* pathway .

**References**

1. Qi M, Nelson KE, Daugherty SC, Nelson WC, Hance IR, et al. (2007) Genomic differences between *Fibrobacter succinogenes* S85 and *Fibrobacter intestinalis* DR7 identified by suppression subtractive hybridization. Appl Environ Microbiol 74: 987-993.

2. Hiratsuka T, Furihata K, Ishikawa J, Yamashita H, Itoh N, et al. (2008) An alternative menaquinone biosynthetic pathway operating in microorganisms. Science 321: 1670-1673.
