# Supplementary material for: The Complete Genome Sequence of Fibrobacter succinogenes S85 Reveals a Cellulolytic and Metabolic Specialist
Source: PLoS One. 2011 Apr 19;6(4):e18814. doi: 10.1371/journal.pone.0018814 (PMC3079729; doi:10.1371/journal.pone.0018814)
Supplement: Text S5 — Transporters. (DOC) [file pone.0018814.s009.doc]

**Text S5: Transporters**

*F. succinogenes* contains transporters for the uptake of a number of substrates (Figure 2), including sulfate, phosphate, and oligopeptides. Sulfate uptake is driven by both ABC and low-affinity transporters, while phosphate transport is mediated by a high-affinity PST system (*phoU-pstBACS*, Fisuc_1702-1705 and Fisuc_1707). Peptide transport is accomplished using the ABC transporter system, and a number of genes encode for this process (Fisuc_0109-0110, Fisuc_043.7, Fisuc_1226-1230, and Fisuc_2985). In addition, the *F. succinogenes* genome encodes for the uptake of iron, including the ferrous iron uptake system (Fisuc_1674-1676), an iron ABC transporter (Fisuc_0194 and Fisuc_0195), and the TonB-ExbB-ExbD system (Fisuc_1733-Fisuc_1738). Other transport systems utilized by *F. succinogenes* include the acquisition of potassium via the low-affinity Trk symporter (Fisuc_1488 and Fisuc 1489); magnesium using the CorA transport system (Fisuc_0857); calcium through a sodium-calcium antiporter (Fisuc_2981); and cobalt through an ABC transporter (Fisuc_1686 and Fisuc_1687).

*F. succinogenes* also possesses a suite of transporters that includes ATP-binding Cassette (ABC) transporters, channel proteins, symporters, and P-type ATPase transporters (Figure 2). Based on previous reports, one or more of these symporters are likely responsible for the transport of glucose and cellobiose into the cell. Glucose uptake by *Fibrobacter succinogenes* S85 has been shown to be inhibited by compounds that interfere with electron transport systems, maintenance of proton or metal ion gradients, or ATP synthesis . Later work demonstrated that *F. succinogenes* uses a pH-sensitive sodium symport mechanism to take up glucose and cellobiose . Glucose and cellobiose uptake and metabolism appear to be strain-dependent, with *F. succinogenes* using both at the same rate when supplied independently and simultaneously when supplied together; two other strains showed different performance . The exact genes responsible for glucose and cellobiose transport cannot be clearly identified from the genome sequence.

**References**

1. Franklund CV, Glass TL (1987) Glucose uptake by the cellulolytic ruminal anaerobe *Bacteroides succinogenes*. J Bacteriol 169: 500-506.

2. Chow JM, Russell JB (1992) Effect of pH and monensin on glucose transport by *Fibrobacter succinogenes*, a cellulolytic ruminal bacterium. Appl Environ Microbiol 58: 1115-1120.

3. Maas L, Glass TL (1991) Cellobiose uptake by the cellulolytic ruminal anaerobe *Fibrobacter* (*Bacteroides*) *succinogenes*. Can J Microbiol 37: 141-147.

4. Gaudet G, Chen KJ (1990) Use of glucose and cellobiose by 3 strains of *Fibrobacter succinogenes*. Reprod Nutr Dev Suppl 2: 201s-202s.
