# Supplementary material for: The Complete Genome Sequence of Fibrobacter succinogenes S85 Reveals a Cellulolytic and Metabolic Specialist
Source: PLoS One. 2011 Apr 19;6(4):e18814. doi: 10.1371/journal.pone.0018814 (PMC3079729; doi:10.1371/journal.pone.0018814)
Supplement: Text S6 — Antibiotic production and resistance. (DOC) [file pone.0018814.s010.doc]

**Text S6: Antibiotic production and resistance**

We analyzed the *F. succinogenes* genome for the presence of genes encoding for enzymes involved in the biosynthesis of secondary metabolites with antibiotic properties but did not find evidence for any such gene clusters. This finding is similar to reports for other ruminal bacterial genomes such as *R. flavefaciens* FD-1 , *P. ruminicola* 23 , and Butyrivibrio proteoclasticus B316 , which do not contain large numbers of genes involved in antibiotic production. This may indicate that few of the known rumen bacteria produce antibiotics or other similar secondary metabolites. Because antibiotic production by bacteria is typically viewed as a way for microbial communities to mediate competition, prevent invasion by non-native bacteria, or to help in establishment of invading bacteria , it is possible that the specialized rumen environment limits the establishment of invading bacteria. Furthermore, microbes in the rumen are thought to be dependent on each other for the production of essential nutrients and may obviate the need for antibiotic production as a mechanism for competition.

We also looked for the presence of antibiotic resistance genes in *F. succinogenes* and found a glyoxylase family resistance gene (Fisuc_1449), a nitroimidazole resistance gene (Fisuc_2301), and a number of β-lactamases (Fisuc_0318, Fisuc_0708, Fisuc_0763, Fisuc_1234, Fisuc_1246, Fisuc_1382, and Fisuc_1616). Other genes putatively involved in antibiotic resistance include general transporters such as the resistance-nodulation-cell division (RND) multidrug transporters (Fisuc_0004, Fisuc_0115, Fisuc_0480, Fisuc_0153, Fisuc_1684, Fisuc_1789, and Fisuc_2028) and the major facilitator superfamily (MFS) transporters (Fisuc_0762 and Fisuc_0802). We also identified a number of genes involved in toxic molecule resistance including 2 acriflavin resistance genes (Fisuc_0290 and Fisuc_0479), a methyl viologen resistance gene (Fisuc_1215), and a camphor resistance gene (Fisuc_1950). Given that *F. succinogenes* is extremely sensitive in vitro to tetracycline, erythromycin, and chloramphenicol, while somewhat less resistant to kanamycin (data not shown), the low diversity of antibiotic and toxic molecule resistance genes may reflect a low incidence of encountering these compounds in the rumen.

**References**

1. Berg Miller ME, Antonopoulos DA, Rincon MT, Band M, Bari A, et al. (2009) Diversity and strain specificity of plant cell wall degrading enzymes revealed by the draft genome of *Ruminococcus flavefaciens* FD-1. PLoS ONE 4: e6650.

2. Purushe J, Fouts D, Morrison M, White B, Mackie R, et al. (2010) Comparative genome analysis of *Prevotella ruminicola* and *Prevotella bryantii*: insights into their environmental niche. Microb Ecol doi: 10.1007/s00248-00010-09692-00248.

3. Kelly WJ, Leahy SC, Altermann E, Yeoman CJ, Dunne JC, et al. (2010) The glycobiome of the rumen bacterium *Butyrivibrio proteoclasticus* B316 highlights adaptation to a polysaccharide-rich environment. PLoS ONE 5: e11942.

4. Wiener P (1996) Experimental studies on the ecological role of antibiotic production in bacteria. Evol Ecol 10: 405-421.

5. Flint HJ, Bayer EA, Rincon MT, Lamed R, White BA (2008) Polysaccharide utilization by gut bacteria: potential for new insights from genomic analysis. Nat Rev Micro 6: 121-131.
