# Supplementary material for: The Complete Genome Sequence of Fibrobacter succinogenes S85 Reveals a Cellulolytic and Metabolic Specialist
Source: PLoS One. 2011 Apr 19;6(4):e18814. doi: 10.1371/journal.pone.0018814 (PMC3079729; doi:10.1371/journal.pone.0018814)
Supplement: Text S7 — DNA Repair. (DOC) [file pone.0018814.s011.doc]

**Text S7: DNA Repair**

DNA repair is an essential process encoded by bacterial genomes that helps to maintain the integrity and viability of genetic information. *F. succinogenes* appears to be extremely sensitive to ultraviolet light, and cells do not survive even very brief exposure to UV sources (data not shown). UV light is known to adversely affect DNA by causing damage through the formation of pyrimidine dimers. Many bacteria have repair mechanisms for reversing this damage, and we searched the *F. succinogenes* genome for genes that may be missing from these pathways. In general, bacterial DNA repair mechanisms can be divided into 5 distinct pathways including direct damage reversal, base excision repair, nucleotide excision repair, mismatch repair, and recombinational repair . Our analysis did not reveal any genes missing from these known pathways involved in DNA repair except for the gene encoding MutM, a pyrimidine glycosylase involved in base excision repair. Furthermore, we did not find any genes putatively encoding a photolyase, which is known to reverse the effects of pyrimidine dimerization , and thus the acute sensitivity displayed by *F. succinogenes* cells could be attributable to the lack of this gene and a gene encoding MutM in the genome. Other sequenced ruminal bacteria genomes like *R. flavefaciens* FD-1 and *P. ruminicola* 23 also lack genes encoding for a photolyase, suggesting that sensitivity to UV light exposure may be a general feature of bacteria inhabiting the perpetually dark ruminal environment.

**References**

1. Aravind L, Walker DR, Koonin EV (1999) Conserved domains in DNA repair proteins and evolution of repair systems. Nucleic Acids Res 27: 1223-1242.

2. Essen L, Klar T (2006) Light-driven DNA repair by photolyases. Cell Mol Life Sci 63: 1266-1277.

3. Berg Miller ME, Antonopoulos DA, Rincon MT, Band M, Bari A, et al. (2009) Diversity and strain specificity of plant cell wall degrading enzymes revealed by the draft genome of *Ruminococcus flavefaciens* FD-1. PLoS ONE 4: e6650.

4. Purushe J, Fouts D, Morrison M, White B, Mackie R, et al. (2010) Comparative genome analysis of *Prevotella ruminicola* and *Prevotella bryantii*: insights into their environmental niche. Microb Ecol doi: 10.1007/s00248-00010-09692-00248.
