# Supplementary material for: The Complete Genome Sequence of Fibrobacter succinogenes S85 Reveals a Cellulolytic and Metabolic Specialist
Source: PLoS One. 2011 Apr 19;6(4):e18814. doi: 10.1371/journal.pone.0018814 (PMC3079729; doi:10.1371/journal.pone.0018814)
Supplement: Text S8 — CRISPRs, insertion sequences, and genomic islands. (DOC) [file pone.0018814.s012.doc]

**Text S8: CRISPRs, insertion sequences, and genomic islands**

Recent work in bacterial genome biology has revealed the presence of an RNAi-like system that acts to provide resistance to phage infection. Clustered regularly interspaced short palindrome repeats (CRISPRs) are found in a number of bacterial genomes and consist of tandem repeats that interact with CRISPR-proteins and can provide immunity against specific phages . We analyzed the *F. succinogenes* genome for the presence of CRISPRs using the web server CRISPRFinder and identified 6 of these elements (Table 1). All 6 CRISPRs correspond to 2 distinct regions on the *F. succinogenes* genome and are found in conjunction with genes that encode for CRISPR-associated proteins that likely interact with these CRISPRs. Specifically, a 2,376 bp CRISPR containing a 48 bp direct repeat and 30 spacers was found downstream of 3 CRISPR-associated proteins (Fisuc_0140-Fisuc_0142). Five other CRISPRs were found from coordinates 1,894,255 – 1,905,597 surrounding 3 other CRISPR-associated genes (Fisuc 1552, Fisuc_1553, and Fisuc_1556). While no *F. succinogenes*-specific phages have been reported, the existence of these CRISPRs in the genome suggests that, like other ruminal bacteria , *F. succinogenes* is also subject to phage infection. We also looked for evidence of insertion sequences in the *F. succinogenes* genome and found 4 of these (Table 2). These include 2 IS231K and 2 IS91 insertion sequences which were found to have closest matches to IS elements in *Bacillus cereus* and *Azoarcus* sp.

Finally, we analyzed the genomic islands predicted to be found in *F. succinogenes* genome as identified from the IslandViewer database (Table 3). Genomic islands are thought to be integrative elements that exhibit abnormal base usage relative to the rest of the genome, encode for an integrase, and are typically found at tRNA loci . These regions are thought to be acquired through the action of bacteriophages, transposons, integrons, and horizontal gene transfer. Genomic islands were first studied in relation to virulence factors (so-called “pathogenicity islands”), but later work has established that these islands can contain a diverse array of genes associated with many processes such as metabolism and degradation . A total of 26 genomic islands were identified in the *F. succinogenes* genome, and many of these islands contain genes involved in metabolism and defense (Table S5). For example, genomic island 5, 15, and 21 contains numerous glycosyl transferases that may be involved in carbohydrate metabolism. Genomic island 11 contains the CRISPR-associated cas proteins associated with phage immunity, and *F. succinogenes* may have acquired this island to resist specific phages present in the rumen.

CRISPR discovery, insertion sequence identification, and genomic island analysis was performed using CRISPRFinder , the ISFinder database (http://www-is.biotoul.fr/, accessed: 10/18/2010), and the IslandViewer database respectively. For CRISPRFinder, all default parameters were used. For IS Finder, all default parameters were used except for an e-value of 1e-05. For IslandViewer, we analyzed the genomic islands predicted from this database for *F. succinogenes*.

**References**

1. Sorek R, Kunin V, Hugenholtz P (2008) CRISPR-a widespread system that provides acquired resistance against phages in bacteria and archaea. Nat Rev Micro 6: 181-186.

2. Grissa I, Vergnaud G, Pourcel C (2007) The CRISPRdb database and tools to display CRISPRs and to generate dictionaries of spacers and repeats. BMC Bioinformatics 8: 172.

3. Klieve A, Bain P, Yokoyama M, Ouwerkerk D, Forster R, et al. (2004) Bacteriophages that infect the cellulolytic ruminal bacterium *Ruminococcus albus* AR67. Lett Appl Microbiol 38: 333-338.

4. Langille MGI, Brinkman FSL (2009) IslandViewer: an integrated interface for computational identification and visualization of genomic islands. Bioinformatics 25: 664-665.

5. Boyd EF, Almagro-Moreno S, Parent MA (2009) Genomic islands are dynamic, ancient integrative elements in bacterial evolution. Trends Microbiol 17: 47-53.

**Table 1**. Identified CRISPRs in the *Fibrobacter succinogenes* S85 genome.

| **Name** | **Start** | **End** | **Length** | **Direct Repeat Consensus Sequence** | **DR Length** | **# of Spacers** |
| --- | --- | --- | --- | --- | --- | --- |
| CRISPR_ 1 | 165662 | 168038 | 2376 | GTTGTTATTGCACTAGTAAAACTAGAAAATCTGAAAGCAATTCACAAC | 48 | 30 |
| CRISPR_ 2 | 1895255 | 1895834 | 579 | CCCTGAAAAGCATTACTCTCGCAAGAGAGAT | 31 | 7 |
| CRISPR_ 3 | 1896753 | 1897007 | 254 | CCCTGAAAAGCATTACTCTCGCAAGAGAGAT | 31 | 3 |
| CRISPR_ 4 | 1902105 | 1902768 | 663 | CCCTGAAAAGCATTCCTCTCGCAAGAGAGATTAAGAC | 37 | 8 |
| CRISPR_ 5 | 1903817 | 1904406 | 589 | CCCTGAAAAGCATTCCTCTCGCAAGAGAGATTAAGAC | 37 | 7 |
| CRISPR_ 6 | 1905308 | 1905597 | 289 | CCCTGAAAAGCATTCCTCTCGCAAGAGAGATTAAGAC | 37 | 3 |

**Table 2**. Identified insertion sequences (IS) in the *Fibrobacter succinogenes* S85 genome.

| **Locus** | **IS** | **Family** | **Group** | **Organism of Top Match** | **Bit score** | **e-value** |
| --- | --- | --- | --- | --- | --- | --- |
| Fisuc_0603 | IS231K | IS4 | IS231 | *Bacillus cereus* | 46 | 2e-06 |
| Fisuc_2199 | IS231K | IS4 | IS 231 | *Bacillus cereus* | 47 | 1e-6 |
| Fisuc_2286 | IS91 | - | - | *Azoarcus sp.* | 81 | 1e-16 |
| Fisuc_3107 | IS91 | - | - | *Azoarcus sp.* | 105 | 4e-24 |

**Table 3.** Genomic islands in the *Fibrobacter succinogenes* S85 genome.

| **Genomic Island** | **Coordinates** | **Locus** | **Product** |
| --- | --- | --- | --- |
| **Genomic Island 1** | 78486..79364 | Fisuc_0069 | DNA methylase N-4/N-6 domain protein |
|  | 79348..79983 | Fisuc_0070 | hypothetical protein |
|  | 79961..80197 | Fisuc_0071 | transcriptional regulator, XRE family |
|  | 80453..80977 | Fisuc_0072 | ErfK/YbiS/YcfS/YnhG family protein |
|  | 80998..81675 | Fisuc_0073 | hypothetical protein |
|  | 81681..82109 | Fisuc_0074 | hypothetical protein |
|  | 82121..82558 | Fisuc_0075 | GCN5-related N-acetyltransferase |
|  | 82555..83409 | Fisuc_0076 | hypothetical protein |
| **Genomic island 2** | 89811..90554 | Fisuc_0086 | hypothetical protein |
|  | 90805..91002 | Fisuc_0087 | hypothetical protein |
|  | 91186..91893 | Fisuc_0088 | protein of unknown function DUF88 |
|  | 92296..92967 | Fisuc_0089 | hypothetical protein |
|  | 93019..94647 | Fisuc_0090 | anaerobic ribonucleoside-triphosphate reductase |
|  | 94746..95348 | Fisuc_0091 | hypothetical protein |
|  | 96713..97396 | Fisuc_0093 | von Willebrand factor type A |
|  | 97398..98399 | Fisuc_0094 | hypothetical protein |
|  | 98440..99126 | Fisuc_0095 | hypothetical protein |
|  | 99130..102276 | Fisuc_0096 | hypothetical protein |
| **Genomic island 3** | 1157598..1157900 | Fisuc_0927 | hypothetical protein |
|  | 1157938..1158255 | Fisuc_0928 | hypothetical protein |
|  | 1158435..1158896 | Fisuc_0929 | hypothetical protein |
|  | 1159106..1160362 | Fisuc_0930 | DNA-cytosine methyltransferase |
|  | 1160434..1161255 | Fisuc_0931 | hypothetical protein |
|  | 1161389..1162093 | Fisuc_0932 | hypothetical protein |
|  | 1162090..1162326 | Fisuc_0933 | transcriptional regulator, XRE family |
|  | 1162465..1162779 | Fisuc_0934 | hypothetical protein |
|  | 1162858..1163802 | Fisuc_0935 | hypothetical protein |
|  | 1163810..1164454 | Fisuc_0936 | hypothetical protein |
|  | 1164508..1164816 | Fisuc_0937 | hypothetical protein |
|  | 1165171..1165620 | Fisuc_0938 | hypothetical protein |
|  | 1165580..1165996 | Fisuc_0939 | transcriptional regulator, XRE family |
| **Genomic island 4** | 1178194..1180320 | Fisuc_0952 | Excinuclease ATPase subunit-like protein |
|  | 1180302..1181711 | Fisuc_0953 | Radical SAM domain protein |
|  | 1182448..1182804 | Fisuc_0954 | hypothetical protein |
|  | 1182891..1183112 | Fisuc_0955 | hypothetical protein |
|  | 1183109..1183513 | Fisuc_0956 | PilT protein domain protein |
|  | 1184225..1184461 | Fisuc_0958 | hypothetical protein |
| **Genomic island 5** | 1187694..1188788 | Fisuc_0962 | NAD-dependent epimerase/dehydratase |
|  | 1188870..1189652 | Fisuc_0963 | glycosyl transferase, WecB/TagA/CpsF family |
|  | 1189653..1191428 | Fisuc_0964 | Haloacid dehalogenase domain protein hydrolase |
|  | 1191444..1192592 | Fisuc_0965 | hypothetical protein |
|  | 1192748..1194172 | Fisuc_0966 | polysaccharide biosynthesis protein |
|  | 1194179..1195360 | Fisuc_0967 | CDP-glycerol:poly(glycerophosphate) glycerophosphotransferase |
|  | 1195357..1196655 | Fisuc_0968 | hypothetical protein |
|  | 1196670..1197080 | Fisuc_0969 | glycerol-3-phosphate cytidylyltransferase |
|  | 1197104..1198027 | Fisuc_0970 | glycosyl transferase family 2 |
|  | 1198059..1198889 | Fisuc_0971 | glycosyl transferase family 2 |
| **Genomic island 6** | 1292291..1294921 | Fisuc_1048 | helicase domain protein |
|  | 1294924..1302150 | Fisuc_1049 | hypothetical protein |
| **Genomic island 7** | 1315633..1316850 | Fisuc_1062 | hypothetical protein |
|  | 1316957..1317634 | Fisuc_1063 | hypothetical protein |
|  | 1317756..1318895 | Fisuc_1064 | hypothetical protein |
|  | 1318931..1319068 | Fisuc_1065 | hypothetical protein |
|  | 1319071..1320120 | Fisuc_1066 | hypothetical protein |
| **Genomic island 8** | 1596244..1596723 | Fisuc_1291 | hypothetical protein |
|  | 1596720..1596935 | Fisuc_1292 | hypothetical protein |
|  | 1597040..1599265 | Fisuc_1293 | protein of unknown function DUF262 |
|  | 1599513..1599893 | Fisuc_1294 | hypothetical protein |
|  | 1599927..1600577 | Fisuc_1295 | transcriptional regulator, XRE family |
|  | 1600527..1603238 | Fisuc_1296 | hypothetical protein |
|  | 1603252..1605765 | Fisuc_1297 | DNA primase small subunit |
|  | 1605769..1607688 | Fisuc_1298 | Site-specific DNA-methyltransferase (adenine-specific) |
|  | 1607735..1610431 | Fisuc_1299 | hypothetical protein |
|  | 1610607..1611929 | Fisuc_1301 | putative ATPase |
|  | 1612149..1613324 | Fisuc_1302 | hypothetical protein |
| **Genomic island 9** | 1596244..1596723 | Fisuc_1291 | hypothetical protein |
|  | 1596720..1596935 | Fisuc_1292 | hypothetical protein |
|  | 1597040..1599265 | Fisuc_1293 | protein of unknown function DUF262 |
|  | 1599513..1599893 | Fisuc_1294 | hypothetical protein |
|  | 1599927..1600577 | Fisuc_1295 | transcriptional regulator, XRE family |
|  | 1600527..1603238 | Fisuc_1296 | hypothetical protein |
|  | 1603252..1605765 | Fisuc_1297 | DNA primase small subunit |
|  | 1605769..1607688 | Fisuc_1298 | Site-specific DNA-methyltransferase (adenine-specific) |
|  | 1607735..1610431 | Fisuc_1299 | hypothetical protein |
|  | 1610607..1611929 | Fisuc_1301 | putative ATPase |
|  | 1612149..1613324 | Fisuc_1302 | hypothetical protein |
| **Genomic island 10** | 1656440..1657513 | Fisuc_1337 | protein of unknown function DUF1016 |
|  | 1657515..1657625 | Fisuc_1338 | hypothetical protein |
|  | 1658216..1658593 | Fisuc_1340 | hypothetical protein |
|  | 1658840..1660768 | Fisuc_1341 | hypothetical protein |
|  | 1660780..1661346 | Fisuc_1342 | hypothetical protein |
|  | 1661520..1662041 | Fisuc_1343 | hypothetical protein |
|  | 1662028..1662543 | Fisuc_1344 | hypothetical protein |
|  | 1662625..1663851 | Fisuc_1345 | restriction modification system DNA specificity domain protein |
| **Genomic island 11** | 1897802..1898833 | Fisuc_1552 | CRISPR-associated protein Cas1 |
|  | 1898830..1899105 | Fisuc_1553 | CRISPR-associated protein Cas2 |
|  | 1907678..1908106 | Fisuc_1554 | hypothetical protein |
|  | 1908123..1908467 | Fisuc_1555 | hypothetical protein |
| **Genomic island 12** | 1959349..1960272 | Fisuc_1602 | hypothetical protein, TIGR02147 |
|  | 1960269..1962482 | Fisuc_1603 | hypothetical protein |
|  | 1962495..1964630 | Fisuc_1604 | hypothetical protein |
| **Genomic island 13** | 2046338..2047246 | Fisuc_1666 | hypothetical protein |
|  | 2047233..2050877 | Fisuc_1667 | hypothetical protein |
|  | 2050877..2052031 | Fisuc_1668 | cysteine desulfurase family protein |
|  | 2052043..2054274 | Fisuc_1669 | type III restriction protein res subunit |
|  | 2054267..2054434 | Fisuc_1670 | hypothetical protein |
|  | 2054416..2056410 | Fisuc_1671 | hypothetical protein |
|  | 2056423..2056950 | Fisuc_1672 | hypothetical protein |
|  | 2056950..2058080 | Fisuc_1673 | putative RNA polymerase, sigma 70 family subunit |
| **Genomic island 14** | 2259371..2263018 | Fisuc_1825 | Putative endonuclease, Z1 domain protein |
|  | 2263021..2264007 | Fisuc_1826 | hypothetical protein |
|  | 2264016..2265662 | Fisuc_1827 | DNA methylase N-4/N-6 domain protein |
|  | 2265674..2268355 | Fisuc_1828 | type III restriction protein res subunit |
|  | 2268554..2270791 | Fisuc_1829 | cell divisionFtsK/SpoIIIE |
|  | 2270858..2272399 | Fisuc_1830 | Heat shock protein 70 |
|  | 2272418..2273137 | Fisuc_1831 | hypothetical protein |
|  | 2273151..2274059 | Fisuc_1832 | hypothetical protein |
|  | 2274339..2274794 | Fisuc_1833 | nucleic acid binding protein |
|  | 2274816..2275109 | Fisuc_1834 | addiction module antitoxin, RelB/DinJ family |
|  | 2275247..2275858 | Fisuc_1835 | hypothetical protein |
|  | 2275914..2276249 | Fisuc_1836 | hypothetical protein |
|  | 2276215..2276457 | Fisuc_1837 | hypothetical protein |
| **Genomic island 15** | 2528526..2530250 | Fisuc_2044 | hypothetical protein |
|  | 2530276..2531043 | Fisuc_2045 | glycosyltransferase sugar-binding region containing DXD motif |
|  | 2531055..2531921 | Fisuc_2046 | glycosyl transferase family 2 |
|  | 2532096..2533853 | Fisuc_2047 | hydrolase (HAD superfamily)-like protein |
|  | 2533856..2534926 | Fisuc_2048 | glycosyl transferase family 2 |
|  | 2534929..2535846 | Fisuc_2049 | hypothetical protein |
|  | 2535848..2536603 | Fisuc_2050 | hypothetical protein |
|  | 2536648..2537607 | Fisuc_2051 | polysaccharide pyruvyl transferase |
|  | 2537604..2538713 | Fisuc_2052 | Radical SAM domain protein |
|  | 2538717..2539484 | Fisuc_2053 | hypothetical protein |
|  | 2539481..2541010 | Fisuc_2054 | polysaccharide biosynthesis protein |
|  | 2541207..2541428 | Fisuc_2055 | hypothetical protein |
|  | 2541421..2541984 | Fisuc_2056 | hypothetical protein |
|  | 2541975..2542229 | Fisuc_2057 | hypothetical protein |
|  | 2542186..2543106 | Fisuc_2058 | glycosyl transferase family 2 |
| **Genomic island 16** | 2706161..2706886 | Fisuc_2192 | hypothetical protein |
|  | 2706889..2707599 | Fisuc_2193 | von Willebrand factor type A |
|  | 2707799..2709217 | Fisuc_2194 | Sigma 54 interacting domain protein |
|  | 2709434..2709679 | Fisuc_2195 | hypothetical protein |
|  | 2709840..2710334 | Fisuc_2196 | hypothetical protein |
| **Genomic island 17** | 3017983..3019086 | Fisuc_2442 | Endo-1,4-β-xylanase |
|  | 3019035..3019748 | Fisuc_2443 | hypothetical protein |
|  | 3019767..3020264 | Fisuc_2444 | hypothetical protein |
|  | 3020313..3020780 | Fisuc_2445 | hypothetical protein |
|  | 3020782..3021471 | Fisuc_2446 | protein of unknown function DUF955 |
|  | 3021471..3021848 | Fisuc_2447 | transcriptional regulator, XRE family |
|  | 3022464..3025112 | Fisuc_2448 | putative type II restriction enzyme (methylase subunit) |
| **Genomic island 18** | 3111897..3114302 | Fisuc_2519 | lipoprotein |
|  | 3114398..3115135 | Fisuc_2520 | hypothetical protein |
|  | 3115276..3117765 | Fisuc_2521 | lipoprotein |
| **Genomic island 19** | 3198647..3200080 | Fisuc_2583 | hypothetical protein |
|  | 3200137..3201459 | Fisuc_2584 | hypothetical protein |
|  | 3201482..3202771 | Fisuc_2585 | hypothetical protein |
| **Genomic island 20** | 3225710..3228100 | Fisuc_2603 | hypothetical protein |
|  | 3228135..3231932 | Fisuc_2604 | hypothetical protein |
|  | 3231934..3236472 | Fisuc_2605 | hypothetical protein |
| **Genomic island 21** | 3287467..3288573 | Fisuc_2652 | glycosyl transferase group 1 |
|  | 3288570..3289610 | Fisuc_2653 | glycosyl transferase group 1 |
|  | 3289597..3290793 | Fisuc_2654 | hypothetical protein |
|  | 3290799..3291953 | Fisuc_2655 | glycosyl transferase group 1 |
|  | 3291973..3292935 | Fisuc_2656 | glycosyl transferase family 2 |
|  | 3292978..3293988 | Fisuc_2657 | hypothetical protein |
|  | 3293989..3294540 | Fisuc_2658 | 4Fe-4S ferredoxin iron-sulfur binding domain protein |
|  | 3294591..3295622 | Fisuc_2659 | glycosyl transferase family 2 |
| **Genomic island 22** | 3298658..3299740 | Fisuc_2663 | hypothetical protein |
|  | 3299742..3300245 | Fisuc_2664 | Fe-S type hydro-lyase tartrate/fumarate beta region |
|  | 3300242..3301579 | Fisuc_2665 | MmgE/PrpD family protein |
|  | 3301672..3302769 | Fisuc_2666 | Capsule synthesis protein, CapA |
|  | 3302766..3303869 | Fisuc_2667 | hypothetical protein |
| **Genomic island 23** | 3309846..3310064 | Fisuc_2673 | hypothetical protein |
|  | 3310096..3311958 | Fisuc_2674 | hypothetical protein |
|  | 3312014..3313024 | Fisuc_2675 | hypothetical protein |
|  | 3313031..3313846 | Fisuc_2676 | glycosyl transferase group 1 |
|  | 3314005..3315081 | Fisuc_2677 | hypothetical protein |
|  | 3315081..3316097 | Fisuc_2678 | hypothetical protein |
|  | 3316102..3317058 | Fisuc_2679 | glycosyl transferase family 2 |
|  | 3317123..3318106 | Fisuc_2680 | glycosyl transferase group 1 |
|  | 3318112..3319242 | Fisuc_2681 | UDP-N-acetylglucosamine 2-epimerase |
|  | 3319267..3320121 | Fisuc_2682 | dTDP-4-dehydrorhamnose reductase |
|  | 3320134..3321174 | Fisuc_2683 | UDP-glucose 4-epimerase |
|  | 3321175..3322404 | Fisuc_2684 | glycosyl transferase group 1 |
| **Genomic island 24** | 3325401..3328982 | Fisuc_2690 | hypothetical protein |
|  | 3329079..3329684 | Fisuc_2691 | hypothetical protein |
|  | 3329681..3330964 | Fisuc_2692 | SMC domain protein |
| **Genomic island 25** | 3333482..3334666 | Fisuc_2695 | UDP-N-acetylglucosamine 2-epimerase |
|  | 3335051..3335350 | Fisuc_2696 | hypothetical protein |
|  | 3335505..3336068 | Fisuc_2697 | hypothetical protein |
|  | 3336068..3336559 | Fisuc_2698 | hypothetical protein |
|  | 3336670..3336843 | Fisuc_2699 | hypothetical protein |
|  | 3337058..3337600 | Fisuc_2700 | hypothetical protein |
|  | 3337603..3338010 | Fisuc_2701 | hypothetical protein |
|  | 3338252..3338470 | Fisuc_2702 | hypothetical protein |
|  | 3338596..3340785 | Fisuc_2703 | hypothetical protein |
| **Genomic island 26** | 3365194..3365631 | Fisuc_2724 | hypothetical protein |
|  | 3366034..3367071 | Fisuc_2725 | hypothetical protein |
|  | 3367106..3368062 | Fisuc_2726 | hypothetical protein |
|  | 3368143..3368607 | Fisuc_2727 | hypothetical protein |
|  | 3368719..3369369 | Fisuc_2728 | hypothetical protein |
|  | 3369366..3370310 | Fisuc_2729 | hypothetical protein |
